# Effect of complement 3/5 knockout on renal proteomics landscape after ischemia and reperfusion injury in rats

**DOI:** 10.14814/phy2.71017

**Published:** 2026-07-20

**Authors:** Dinesh Bhattarai, Amod Sharma, Madison McGraw, Se‐Ran Jun, Neelam Joshi, Neriman Gokden, Samuel Mackintosh, Nirmala Parajuli

**Affiliations:** ^1^ Department of Pharmacology and Toxicology University of Arkansas for Medical Science Little Rock Arkansas USA; ^2^ Division of Transplantation & Advanced Hepatobiliary Surgery, Department of Surgery University of Utah Salt Lake City Utah USA; ^3^ Department of Medical Informatics University of Arkansas for Medical Sciences Little Rock Arkansas USA; ^4^ Department of Pathology University of Arkansas for Medical Sciences Little Rock Arkansas USA; ^5^ Department of Biochemistry and Molecular Biology University of Arkansas for Medical Sciences Little Rock Arkansas USA

**Keywords:** acute kidney injury, complement C3, complement C5, ischemia–reperfusion injury, kidney transplantation, proteomics

## Abstract

Ischemia–reperfusion injury (IRI) is a major driver of acute kidney injury and development of chronic kidney disease. Although complement activation worsens IRI, the roles of upstream (C3) versus downstream (C5) components remain unclear. Renal IRI was surgically induced in C3 knockout (C3^−^/^−^) and C5 knockout (C5^−^/^−^) Lewis rats, and the renal function as well as histopathology were systematically assessed. Further, quantitative proteomics coupled with pathway enrichment analysis was performed to define complement‐dependent mechanisms. C3 and C5 deficiency conferred strong protection against renal IRI with improved renal function, reduced tubular necrosis, and lower expression of injury markers (KIM‐1, NGAL). Post‐IRI, C3^−/−^ enhanced mitochondrial, metabolic, and purine pathways while suppressing immune and extra‐cellular matrix programs. C5^−/−^ affected extracellular matrix remodeling and structural pathways with modest immune suppression. We demonstrate for the first time that upstream C3^−/−^ impacts a diverse range of renal injury and repair mechanisms compared to C5^−/−^ alone, although both interventions successfully reduced IRI‐mediated injury. Together, these findings highlight strategies for future complement‐based therapies targeting the upstream or downstream cascade.

## Graphical abstract

Comparative proteomic analysis of kidneys from C3‐/‐ and C5‐/‐ rats following ischemia‐reperfusion injury (IRI) reveals diverse range of renal injury and repair mechanisms. C3 knockout enhanced mitochondrial, metabolic, and purine pathways while suppressing immune and extracellular matrix programs. In contrast, C5 absences affected extracellular matrix remodeling and structural pathways with modest immune suppression.

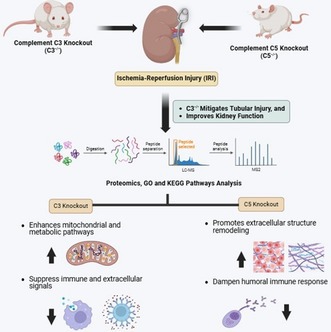

## 1 INTRODUCTION

Chronic kidney disease (CKD) affects more than 850 million people worldwide and represents a major global health burden, contributing substantially to morbidity, mortality, and rising healthcare costs (Bikbov et al., 2020; Li et al., 2025; Mark et al., 2025). The primary risk factor for the development of CKD is a prior acute insult, frequently due to ischemia–reperfusion injury (IRI). This transient interruption and restoration of blood flow induces cellular stress and impairs renal function, contributing to widespread tissue necrosis/inflammation which results in permanent fibrotic changes (Eltzschig & Eckle, 2011; Linkermann et al., 2013). There are currently no clinical treatments available for renal IRI despite its relevance in CKD, exposing a critical need to identify therapeutic targets and strategies.

Renal IRI is characterized by tightly interconnected metabolic and inflammatory processes. Ischemia disrupts mitochondrial function, resulting in depletion of cellular ATP and cell death, while reperfusion exacerbates injury through reactive oxygen species generation and immune activation (Eltzschig & Eckle, 2011; Menke et al., 2014). The complement system, linked to mitochondrial disruption in prior studies (Ishii et al., 2021; Rahman et al., 2021; Tan et al., 2020; Tsai et al., 2019), exacerbates renal IRI through the activation of complement components C3 and C5 which generates effector molecules such as C3a, C5a, and the membrane attack complex (MAC, C5b‐9) (Danobeitia et al., 2014; Situmorang & Sheerin, 2019; Uehara et al., 2018). These mediators promote leukocyte recruitment, cytokine release, and direct tubular injury following ischemia–reperfusion. However, the role(s) of C3 and C5 in renal IRI have yet to be completely defined (Hu et al., 2018).

Importantly, the roles of individual complement components are not uniform. C3 occupies a central upstream position, integrating signals from all activation pathways and regulating complement amplification. Activation of C3 results in the formation of anaphylatoxin mediator C3a as well as C3b, which is crucial for downstream C5 activation. Previous studies show that C3 inhibition or deletion reduces inflammation and renal injury post‐IRI, although its effects on functional recovery are less consistent (Peng et al., 2012; Wu et al., 2022; Zheng et al., 2006). In contrast, C5 acts downstream and is more directly associated with tissue injury through generation of the potent anaphylatoxin C5a and initiation of MAC formation (Arumugam et al., 2003). Both experimental and therapeutic studies targeting C5 demonstrate reduced inflammation, attenuated tubular injury, and improved renal function following IRI (Buelli et al., 2024; de Vries et al., 2003; Mcgraw et al., 2023). These observations suggest that upstream and downstream complement components may differentially regulate renal injury pathways, but this has not been systematically investigated.

Mass spectrometry–based proteomics now offers an opportunity to address this gap by enabling unbiased, system‐level analysis of protein networks. Prior proteomic studies of renal IRI have identified extensive remodeling of mitochondrial metabolism, fatty acid oxidation, oxidative stress responses, cytoskeletal organization, and inflammatory signaling, underscoring the integrated nature of kidney injury and repair (Chen et al., 2023; Li et al., 2024; Luo et al., 2026). Emerging human proteomics data further supports a link between complement activation and global protein remodeling in kidney disease (Kesarwani et al., 2024; Md Dom et al., 2025; Yun et al., 2025). However, despite these advances, proteomic analyses specifically examining complement‐driven mechanisms in renal IRI remain scarce. Notably, no prior study has compared global renal protein expression changes in C3‐ and C5‐deficient models following IRI.

To address this critical gap, we generated a novel C3 knockout (C3^−/−^) Lewis rat model using CRISPR–Cas9 genome editing and leveraged our previously established C5 knockout (C5^−/−^) model (McGraw et al., 2025). We *hypothesized that deficiencies in C3 and C5 will mitigate renal IRI pathophysiology by suppressing pro‐inflammatory pathways and contributing to metabolic adaptation*.

By integrating physiological, histological, immunological, and quantitative proteomic analyses, this study provides a systems‐level understanding of how complement components orchestrate renal injury and recovery. Our findings demonstrate that C3 and C5 regulate distinct yet interconnected molecular programs during ischemic kidney injury, offering new mechanistic insight and identifying potential targets for precision complement‐based therapies.

## 2 MATERIALS AND METHODS

### 2.1 C3 and C5 knockout rat models

C3^−/−^ and C5^−/−^ Lewis rats were generated using CRISPR/Cas9‐mediated genome editing by the University of Michigan Transgenic Core. CRISPR/Cas9 reagents were microinjected into fertilized embryos to induce double‐strand breaks in exon 2 of C3‐201 or exon 3 of C5‐201 transcripts. Base pair deletions were introduced via nonhomologous end‐joining, resulting in frameshift mutations and premature stop codons (Filipiak & Saunders, 2006).

Founder animals (G0) were identified by PCR‐based genotyping of tail snip DNA (DNeasy Blood & Tissue Kit, Qiagen, #69504) using Forward and Reverse primers CTTTCTCAGCTCAAGCAAAGTCTTTATGG and CTTATCTGCATTGAATTCCTTACTGGCTG, respectively. Positive G0 rats were bred with wild‐type females to generate heterozygous G1 offspring. Nine heterozygous pups per line were shipped to the University of Arkansas for colony expansion and experiments.

### 2.2 Animal care and colony maintenance

Heterozygous C3^+/−^ and C5^+/−^ males and females were housed as monogamous breeding pairs during the female estrous cycle. Males were removed after pregnancy confirmation and reintroduced after weaning (~3 weeks). Rats were maintained under a 12‐h light/dark cycle, temperature‐regulated housing, and fed standard chow (LabDiet, #3002906‐704) with water ad libitum. All procedures were approved by the IACUC at the University of Arkansas for Medical Sciences and complied with NIH guidelines.

### 2.3 Genotyping

Genomic DNA from postweaning tail snips (3–5 mm) was amplified using Phire Direct PCR Master Mix (Thermo Fisher, #F170S). PCR conditions: 98°C for 5 min, 40 cycles of 98°C for 5 s and 70°C for 30 s. C3 and C5 deletion yielded 557 bp and 334 bp products, respectively. PCR products were resolved on 1.5% agarose gels (TBE buffer, Invitrogen, 15581‐028) with ethidium bromide (15585‐011) and visualized using iBright 1500 (Invitrogen, #A44241).

### 2.4 Renal ischemia–reperfusion injury

Male and female wild‐type, C3^−/−^ and C5^−/−^ rats (8–10 weeks) were anesthetized with 5% isoflurane (maintenance 2%) and subjected to midline abdominal incision. Both renal pedicles were clamped with atraumatic vascular clamps for 60 min. Right nephrectomy was performed, and the left kidney was re‐perfused. Muscles and skin were closed with sutures and staples. Rats were returned to their cages for recovery.

At 24 h post‐reperfusion, animals were re‐anesthetized for kidney and blood collection. Animals were euthanized by exsanguination. After rinsing in ice‐cold PBS, kidneys were collected for histological examination, and the remaining tissue was snap‐frozen in liquid nitrogen and stored at −80°C for subsequent proteomic and Western blot analyses.

### 2.5 Hematology

Blood was collected into Eppendorf tubes (serum/blood chemistry) or EDTA tubes (complete blood counts). Blood gases and chemistry were measured using VetScan i‐STAT (CG4+ and CHEM8+ cartridges, Abaxis). Complete blood counts were analyzed using VetScan HM5 (Abaxis, #790–0000). Serum was prepared by centrifugation at 350 × g for 10 min.

### 2.6 Protein extraction and Western blotting

Kidneys were homogenized or powdered and lysed in RIPA buffer (Pierce, #89900) with 1.2 mM Na_3_VO_4_, 2.5 mM NaF, 1 mM DTT (Bio‐Rad, #161–0611), 1 mM PMSF (Millipore, #532332), and protease inhibitors (Pierce, #1860932) as previously described (Parajuli et al., 2011). Lysates were centrifuged at 16,000 × g for 20 min at 4°C. Protein concentration was measured with BCA assay (Pierce, #23225). Proteins were separated by SDS‐PAGE and transferred to PVDF membranes. Membranes were stained with Red Alert (Millipore, #71078), blocked with 5% milk in TBS‐T, incubated with primary antibodies, and probed with HRP‐conjugated secondary antibodies (Table 1). Signals were detected with SuperSignal West Pico PLUS (Thermo Fisher, #34580) using iBright 1500 (Invitrogen, #A44241). Densitometry was performed with AlphaEase FC software (Alpha Innotech).

**TABLE 1:** List of the antibodies.

Antibody	Source	Application	Dilution	Catalog number
KIM‐1	LSBio	IHC, WB	1:2000 IHC; 1:1000 WB	LS‐C312791
NGAL	LSBio	IHC	1:2000	LS‐C37211
C3	Abcam	IHC, WB	1:8000 IHC; 1:1000 WB	Ab200999
LAD1	ThermoFisher	WB	1:1000	PA5‐145485
Hsp72	LS Bio	WB	1:1000	LS‐C82983
Neutrophil Elastase	Cell Signaling Technology	IHC	1:400	440,305
CD68	ThermoFischer	IHC	1:200	PA5‐78996
Peroxidase Goat Anti‐Mouse IgG	Jackson Immuno Research	WB	1:30,000	115–035‐166
Peroxidase Goat Anti‐Rabbit IgG	Jackson Immuno Research	WB	1:30,000	111–035‐144

### 2.7 Histology and immunohistochemistry

Formalin‐fixed kidneys were embedded in paraffin; two cross‐sections per kidney (4–5 μm) were mounted on glass slides (Fisher, #12‐544‐3). Sections were deparaffinized, rehydrated, and stained with Periodic acid–Schiff (PAS) (Parajuli et al., 2011). For immunohistochemistry, antigen retrieval was performed in sodium citrate buffer (pH 6.0), followed by quenching (BLOXALL™, Vector, #SP‐600) and blocking with 2.5% goat serum (Lo et al., 2021). Primary antibodies were detected using ImmPRESS™ Anti‐Rabbit (Vector Laboratories, #MP‐7451‐15) or Anti‐Mouse IgG (Vector Laboratories, # MP‐7452‐15) with ImmPACT™ DAB substrate (Vector Laboratories, #SK‐4100). Sections were counterstained with Mayer's Hematoxylin, blued, dehydrated, and mounted with Cytoseal‐60. Images were captured with a Nikon Eclipse E800 microscope, and a semi‐quantitative scoring system was used to assess histopathology. The scoring scale ranged from 0 (no changes), 1 (mild, affecting 1%–25%), 2 (moderate, 26%–50%), to 3 (severe, greater than 50%). Acute tubular necrosis (ATN) scoring was performed by a blinded renal pathologist. Histological/immunohistochemical analyses and scoring were performed on ten randomly selected fields from at least two kidney sections per animal.

### 2.8 Proteomics sample preparation and LC–MS/MS analysis

Kidney tissue samples from male rats were processed to extract total protein, which was then reduced, alkylated, and purified using a chloroform/methanol method (Puopolo et al., 2024). Male rats were selected for this analysis as renal AKI affects male patients significantly more than female patients, and in rodent models female rats are less likely to develop CKD (Curtis, 2024; Lima‐Posada et al., 2017). The purified proteins were digested with sequencing‐grade porcine trypsin (Promega) to generate peptides suitable for mass spectrometry analysis. The resulting peptides were separated using a high‐performance Ion‐Opticks TS column (25 cm × 75 μm, 1.7 μm C18) connected to an EASY‐Spray nano‐source, maintained at 60°C. Before separation, peptides were trapped on a PepMap Neo column (300 μm × 5 mm) using a Vanquish Neo UHPLC system (Thermo Scientific), which kept samples cooled at 11°C. Peptides were eluted with a 35‐min gradient at a flow rate of 0.35 μL/min using two buffers: Buffer A (0.1% formic acid, 0.5% acetonitrile in water) and Buffer B (80% acetonitrile, 20% water, 0.1% formic acid). Eluted peptides were ionized via electrospray (2.5 kV) and analyzed on an Orbitrap Astral mass spectrometer (Thermo Scientific). DIA acquisition was performed using 199 × 3 Th windows across 380–980 Th with 25% HCD collision energy. MS1 scans were recorded at 240,000 resolution, and MS2 fragment scans covered 150–2000 Th.

### 2.9 Quantitative proteomics and functional annotation

After mass spectrometry data acquisition, raw files were processed using Spectronaut v20.1 (Biognosys) and searched against the *UniProt Rattus norvegicus* proteome (Proteome ID: UP000002494, Taxon ID: 10116; 2025 release, version 2) using the directDIA approach. Peptide and protein identifications were filtered to a 1% false discovery rate (FDR) using a target‐decoy approach. Protein quantification was performed in Spectronaut based on MS2‐level precursor peak areas, with protein intensities derived by standard peptide‐to‐protein aggregation. Carbamidomethylation of cysteine was set as a fixed modification, while N‐terminal acetylation and methionine oxidation were included as variable modifications.

To ensure data reliability, protein intensity values were evaluated with ProteiNorm and normalized using variance stabilization normalization (VSN) (Graw et al., 2020; Huber et al., 2002). Differential expression analysis was performed in R using the proteoDA framework, applying linear models from the limma package with empirical Bayes smoothing to stabilize standard errors (Ritchie et al., 2015; Thurman et al., 2023). Proteins exhibiting an FDR‐adjusted *p*‐value <0.05 and a fold change greater than 2 were considered significantly altered.

Significantly altered proteins were subjected to downstream bioinformatic analyses, including functional annotation and pathway enrichment. Gene Ontology (GO) enrichment analysis was performed using the WEB‐based GEne SeT AnaLysis Toolkit (WebGestalt, version 2024) (Liao et al., 2019) and the clusterProfiler R package (version 4.10.1) to identify overrepresented biological processes associated with differentially expressed proteins (Wu et al., 2021).

In addition, Gene Set Enrichment Analysis (GSEA) was conducted using ShinyGO (v.0.85) on ranked protein lists to assess coordinated changes in GO Biological Process terms (Ge et al., 2020). Enrichment scores were normalized to account for gene set size, and significantly enriched pathways were identified based on normalized enrichment scores (NES).

### 2.10 Statistical analysis

Data are presented as mean ± SD. Normality was assessed with Shapiro–Wilk. For normally distributed data, one‐way or two‐way ANOVA with Tukey's post hoc (*α* = 0.05) and Bonferroni's correction. Non‐normal data were analyzed with the Mann–Whitney *U* test. Statistical significance: *p* < 0.05. Graphs were generated using GraphPad Prism v10.

## 3 RESULTS

### 3.1 Generation of C3 ^−/−^ and C5 ^−/−^ rat models

**FIGURE 1:**
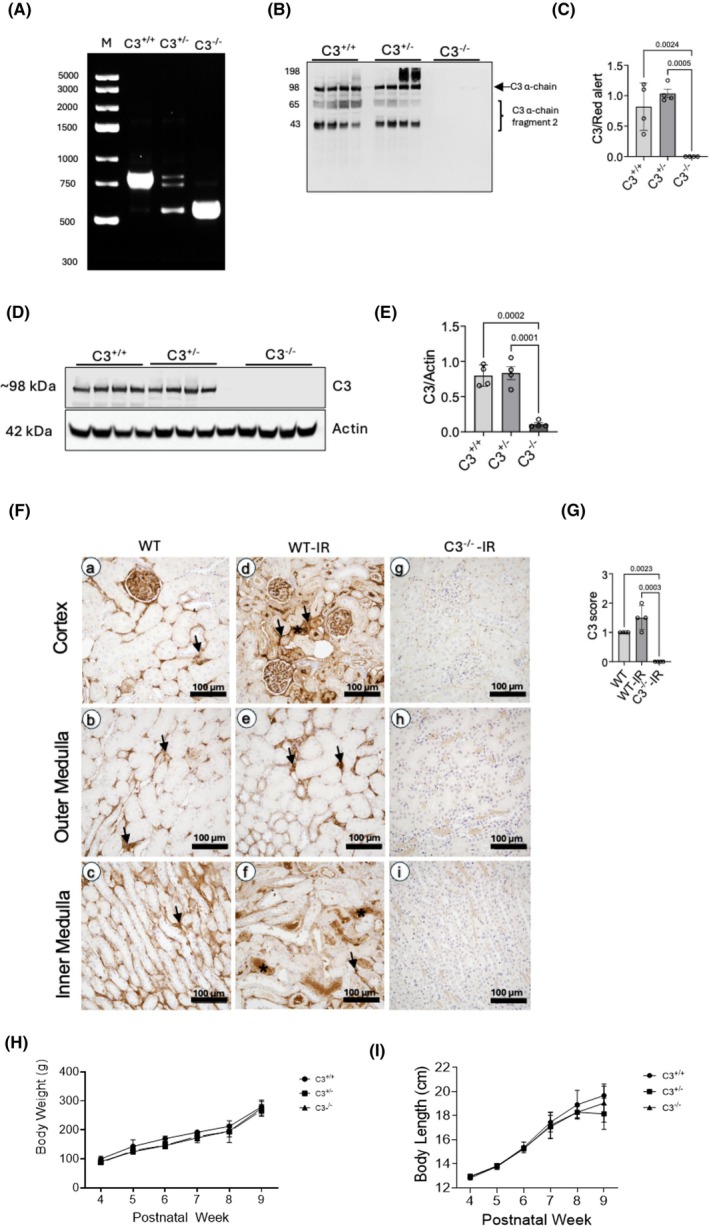
Generation and validation of a global C3 knockout rat model. CRISPR/Cas9 technology was leveraged to induce double‐strand breaks in C3‐201 exon 2 in fertilized rat eggs. Base pair deletion by nonhomologous end‐joining resulted in mRNA with premature stop codons and an inability to form the C3 protein. (A) Representative PCR genotyping of tail DNA showing a single 805‐bp band corresponding to the WT C3^+/+^ allele, a 557‐bp band corresponding to the disrupted C3^−/−^ allele, and both bands in heterozygous (C3^+/−^) rats. A 2 kbp DNA ladder was used as a reference marker (M). (B) Tail vein blood samples collected from C3^+/+^, C3^+/−^, and C3^−/−^ rats were processed for serum SDS‐PAGE Western blotting. C3^−/−^ rats lack the C3 α‐chain (110 kDa) and degraded fragments (68 and 42 kDa). (C) Densitometry analysis of C3 relative to total serum protein as quantified via Red Alert stain reveals the absence of C3 protein. Data are shown as the mean ± SD, *n* = 4. (D) Kidney tissue samples processed for SDS‐PAGE Western blotting show the absence of C3 protein in C3^−/−^ rats. (E) Bar graph depicting densitometry ratio of C3 and Actin. Data are shown as the mean ± SD, *n* = 4. (F) Formalin fixed kidney sections from wild‐type (WT) or C3^−/−^ rats after surgically induced ischemia–reperfusion (IR), immunohistochemically stained for C3 protein. Sham kidneys were used as a control. Black arrows indicate peritubular C3 deposition, and asterisks indicate intratubular deposition of C3 within necrotic tubules. Bar indicates 100 μm. (G) Semi‐quantitative scoring of C3 deposition across experimental groups. Data are shown as the mean ± SD, *n* = 4. (h, i) Animal growth for each genotype, evaluated as (H) body weight and (I) body length recording weekly from postnatal week 4 to postnatal week 9. Data are shown as the mean ± SD, *n* = 4. *p* values for pairwise comparisons are displayed above the corresponding bars.

To investigate the role of complement C3 in renal injury, CRISPR–Cas9 genome editing was used to generate Lewis rats carrying a targeted deletion of the C3 gene. G1 pups (C3^+/−^) were shipped to the University of Arkansas for Medical Sciences to establish the breeding colony and produced a G2 generation of C3^−/−^ rats carrying the full deletion of C3 (100% knockout). Throughout these studies, C3^−/−^ rats are compared with WT (wild‐type) littermates. Genotyping was performed using genomic DNA extracted from tail biopsies, and PCR amplification with C3‐specific primers confirmed the expected genotypes. As shown in Figure 1A, the wild‐type C3^+/+^ allele produced a DNA band at 805 bp, whereas the knockout C3^−/−^ allele generated a band at 557 bp. Heterozygous C3^+/−^ rats displayed both bands.

To verify the global deletion of C3 at the protein level, serum samples from experimental animals were analyzed by Western blotting. The C3 α‐chain (~110 kDa) and degraded fragments (68 and 43 kDa, respectively) were detected in serum from WT and C3^+/−^ rats but were completely absent in C3^−^/^−^ animals, indicating that circulating C3 protein was eliminated in the C3^−/−^ rats (Figure 1B,C). To further assess C3 expression in the kidney, renal tissue lysates were subjected to SDS–PAGE followed by immunoblotting for C3. Consistent with the serum findings, we detected basal C3 protein in kidneys from WT rats which was retained post‐IRI but was completely absent in C3^−/−^ rats (Figure 1D,E). This finding was further dissected using immunohistochemical analysis (Figure 1F,G), which demonstrated a basal level of peritubular C3 staining in WT rats (Figure 1F a–c) and C3 deposition in necrotic tubules post‐IRI (Figure 1F d–f) which was eliminated by C3^−/−^ (Figure 1F g–i). The animal growth rate was not significantly impacted by C3^−/−^ (Figure 1H,I). Together, these findings confirm the successful generation of a global C3 knockout rat model with complete loss of C3 expression in both circulation and kidney tissue. Similarly, our group has previously reported the successful development and characterization of a novel C5^−/−^ rat model (Mcgraw et al., 2023). By utilizing both models, these studies allowed a direct comparison between upstream and downstream complement pathway inhibition in the context of renal IRI.

### 3.2 C3 ^−/−^ improves kidney function and ameliorates necrosis post‐IRI

**FIGURE 2:**
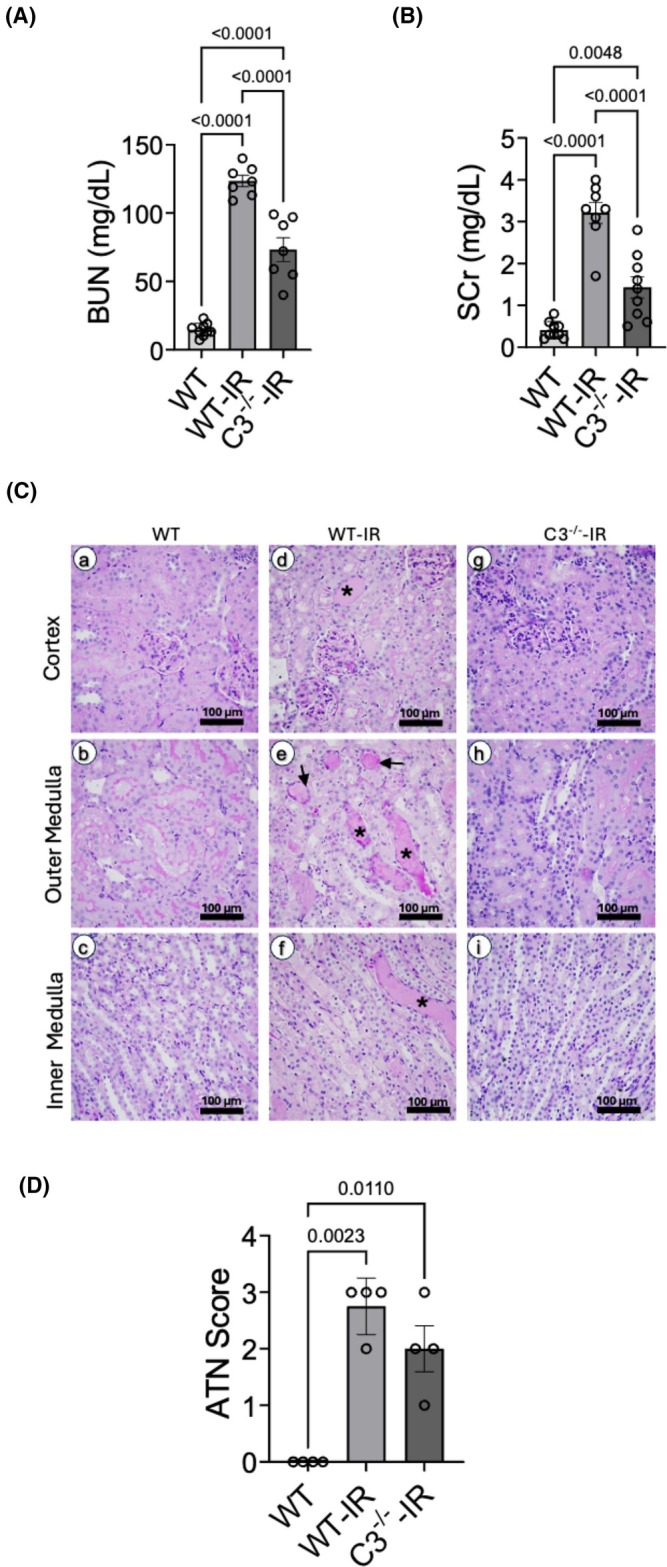
Complement C3 deficiency mitigates renal dysfunction and tubular injury following ischemia–reperfusion (IR). Wild‐type (WT) and homozygous C3 knockout (C3^−/−^) rats underwent bilateral renal ischemia (60 min) plus reperfusion (24 h) injury (IR). (A, B) Bar graphs showing quantification of kidney function biomarkers (A) blood urea nitrogen (BUN) and (B) serum creatinine (SCr), as quantified via the VetScan i‐STAT system. Data are shown as the mean ± SD, *n* = 7–9. (C) Formalin‐fixed kidney sections isolated from rats post‐surgery were stained with Periodic Acid‐Schiff (PAS) to visualize tissue morphology. IR kidneys show signs of early tubular degeneration (black arrows) and necrotic tubules (black asterisks). Bar indicates 100 μm. (D) Semi‐quantitative acute tubular necrosis (ATN) scores (quantified by a blinded pathologist) showing severe tubular injury in WT‐IR rats and significantly reduced injury in C3^−/−^‐IR rats. Data are presented as mean ± SD, *n* = 4. *p* values for pairwise comparisons are displayed above the corresponding bars.

Complement activation is a well‐recognized feature of IRI, with cleaved components of C3 playing a central role in mediating inflammatory and tissue‐damaging responses (Peng et al., 2012; Zheng et al., 2006). To better understand the contribution of C3 to acute kidney injury, we examined renal function and tissue morphology in WT and C3^−/−^ rats following IRI. As anticipated, IRI caused a marked deterioration in kidney function in WT rats, reflected by significant elevations in blood urea nitrogen (BUN) and serum creatinine (SCr) levels (Figure 2A,B). In contrast, C3^−/−^ rats displayed significantly lower levels of both BUN and SCr after injury, indicating that the absence of C3 partially protects against IRI‐induced renal dysfunction.

Having observed improvements in renal function, we next assessed whether C3 deficiency also protects the structural integrity of the kidney. Histological examination revealed clear differences in the extent of renal damage between groups. Compared to WT controls which demonstrated baseline glomerular and tubular morphology (Figure 2C a–c), Periodic Acid‐Schiff (PAS) staining showed extensive tubular injury (black arrows) and necrosis (black asterisks) in WT kidneys following IRI (Figure 2C d–f). In comparison, kidneys from C3^−/−^ rats exhibited relatively preserved tubular architecture, with fewer necrotic regions and less visible structural disruption (Figure 2C g–i). To provide a semi‐quantitative assessment of tissue damage, acute tubular necrosis (ATN) scoring was performed by a blinded, licensed renal pathologist. As expected, the ATN score was markedly elevated in WT rats after IRI compared to healthy controls, which was partially mitigated by C3^−/−^ (Figure 2D). These data supported the protective effect of C3 deficiency and substantiated similar observations in C5^−/−^ rats (McGraw et al., 2025) after ischemic insult.

**FIGURE 3:**
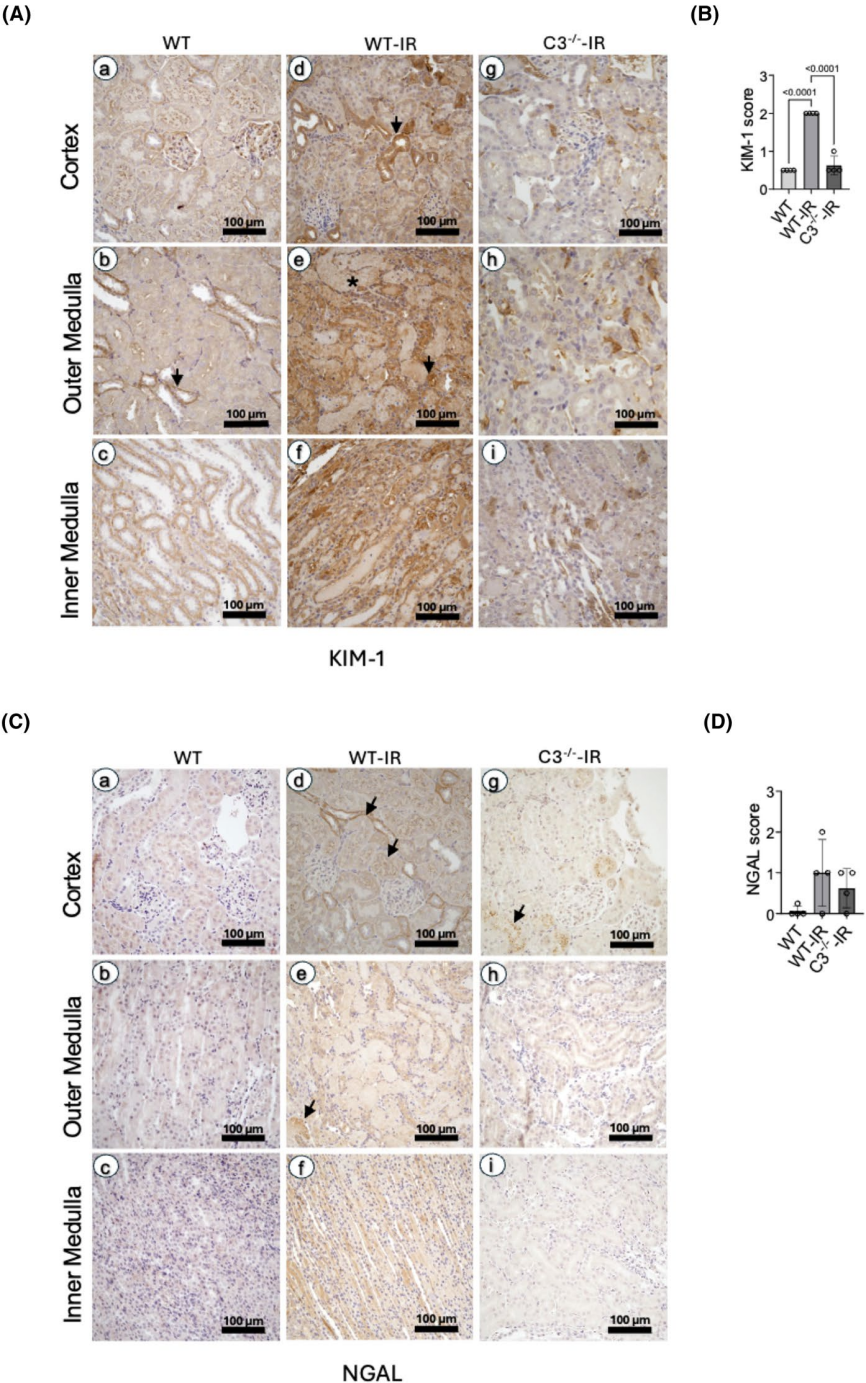
C3 deficiency attenuates tubular injury marker expression following renal ischemia–reperfusion injury. Kidneys were isolated post‐IR and processed for immunohistochemical analysis. (A) Representative micrographs (*n* = 4 per group) of formalin‐fixed kidney sections immunostained for Kidney Injury Molecule‐1 (KIM‐1; black arrows show deposition in tubular/peritubular regions). Black asterisks indicate injured, necrotic tubules. (B) Semi‐quantitative analysis of KIM‐1 deposition across experimental groups. Data are expressed as the mean ± SD, *n* = 4. (C) Representative micrographs (*n* = 4) of formalin‐fixed kidney sections stained for neutrophil gelatinase‐associated lipocalin (NGAL; black arrows). (D) Semi‐quantitative analysis of NGAL deposition across experimental groups. Data are expressed as the mean ± SD, *n* = 4. *p* values for pairwise comparisons are displayed above the corresponding bars.

We also evaluated well‐established molecular markers of kidney injury to further characterize the extent of tubular damage. Immunohistochemical analysis revealed baseline expression of kidney injury molecule‐1 (KIM‐1; black arrows) (Figure 3A a–c) and neutrophil gelatinase‐associated lipocalin (NGAL; black arrows) (Figure 3C a–c) in WT kidneys. KIM‐1 staining was exacerbated following IRI (Figure 3A d–f,B) in WT rats consistent with severe tubular injury and necrosis (black asterisks). KIM‐1 was also markedly reduced in C3^−/−^ rats post‐IRI (Figure 3A g–i,B), consistent with our prior observations that C3^−/−^ confers a protective effect. By contrast, NGAL exhibited no statistically significant changes from its baseline expression following IRI or C3^−/−^, although WT rats exhibited the widest variation in NGAL scores post‐injury (Figure 3C d–i,D). Importantly, these findings also align with our previous work demonstrating that deletion of C5 reduced kidney injury markers after IRI (Mcgraw et al., 2023). Together, these results further highlight the critical contribution of complement activation to the pathogenesis of ischemia‐induced kidney injury.

### 3.3 C3 ^−/−^ does not alter systemic physiology during IRI

**FIGURE 4:**
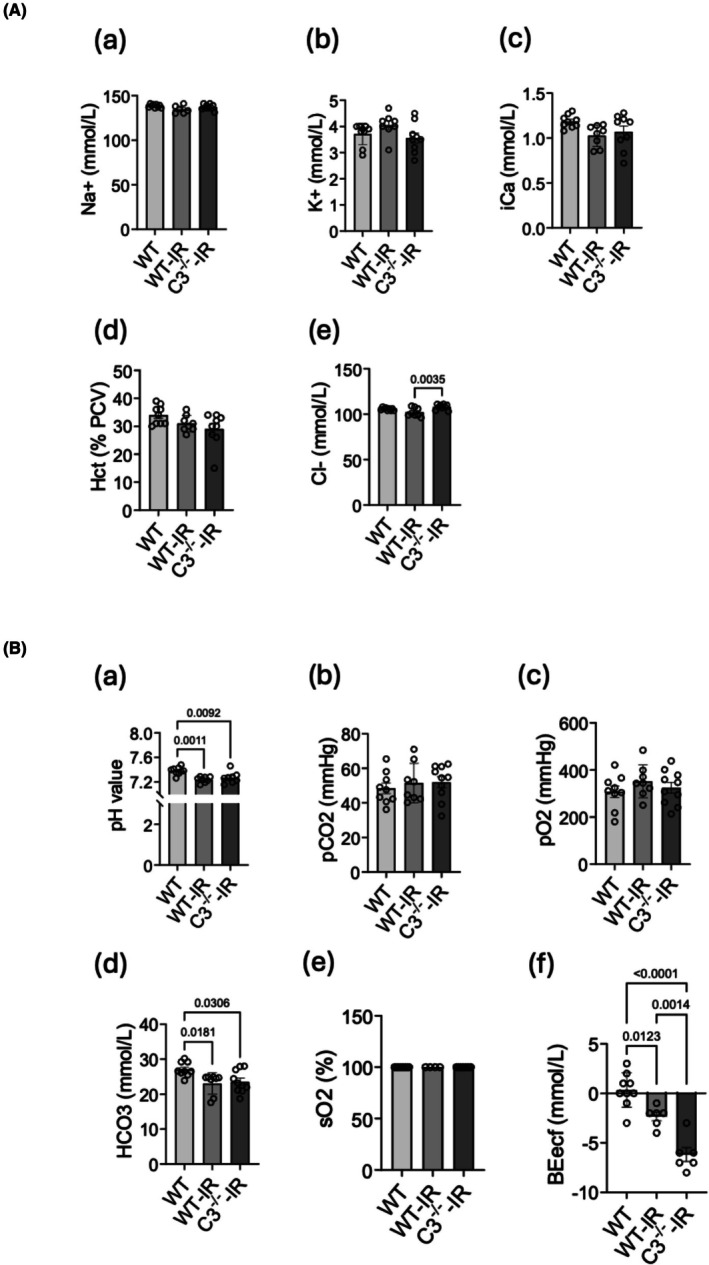
Effects of C3 deficiency on systemic electrolyte balance and acid–base status following renal ischemia–reperfusion injury. Heparinized whole blood was collected from wild‐type (WT) and C3 knockout (C3^−/−^) rats post‐IRI for analysis using the VetScan i‐STAT system. (A) Serum electrolyte parameters including (a) sodium (Na^+^), (b) potassium (K^+^), (c) ionized calcium (iCa), (d) hematocrit (Hct), and (e) chloride (Cl^−^). (B) Arterial blood gas analysis showing (a) pH, (b) partial pressure of carbon dioxide (pCO_2_), (c) partial pressure of oxygen (pO_2_), (d) bicarbonate (HCO_3_^−^), (e) oxygen saturation (sO_2_), and (f) base excess of extracellular fluid (BEecf). Data are presented as the mean ± SD, *n* = 8–10. *p* values for pairwise comparisons are displayed above the corresponding bars.

To ensure that the protective effects of C3 deletion on the kidney were not influenced by systemic physiological changes, we next evaluated whether the absence of C3 alters baseline electrolyte balance or respiratory/metabolic status. Blood samples were collected from all experimental groups and analyzed for standard blood chemistry and arterial blood gas parameters using a veterinary diagnostic analyzer. No significant differences were observed in sodium (Na), potassium (K), ionized calcium (iCa), or hematocrit (Hct) levels among experimental groups (Figure 4A a–d), indicating that neither IRI nor C3^−/−^ altered these hematological parameters. However, C3^−/−^ rats exhibited a significant increase in blood chloride (Cl^‐^) levels compared with WT rats following IRI (Figure 4A e). Previous studies of complement deficiency primarily report alterations in immune and inflammatory responses rather than systemic electrolyte disturbances (Reis et al., 2006; Gorsuch et al., 2012), suggesting that the modest difference in Cl^‐^ may not be physiologically significant. These findings indicate that the improved renal outcomes observed in C3^−/−^ rats following IRI are unlikely to be driven by systemic electrolyte differences.

Renal IRI is commonly associated with metabolic acidosis due to the impairment of acid excretion (Hnin Si et al., 2013). Furthermore, the use of isoflurane anesthesia may result in respiratory depression which can impact experimental results (Taconic Biosciences, 2023). We thus measured arterial blood gas parameters to verify physiological stability, maintain experimental reproducibility, and assess potential effects on IRI‐mediated acidosis. We observed a slight decrease in blood pH in WT rats post‐IRI compared to the healthy baseline, but C3^−/−^ did not significantly affect IRI‐mediated changes to overall pH (Figure 4B a). Respiratory parameters including oxygen saturation (sO_2_), partial pressure of carbon dioxide (pCO_2_), and partial pressure of oxygen (pO_2_) also did not differ among the study groups (Figure 4B b,c,e). By contrast, both bicarbonate (HCO_3_) and the base excess (BEecf) were decreased from their healthy baseline following IRI (Figure 4B d, f), signifying a potential metabolic acidosis or loss of bicarbonate in the extracellular fluid. As C3^−/−^ did not significantly improve HCO_3_ (Figure 4B d) and even further reduced BEecf post‐IRI (Figure 4B f), it is unlikely that C3 is involved in acid–base homeostasis. Combined with the minimal effect of C3^−/−^ on blood chemistry, these results show that C3 deficiency does not meaningfully alter systemic electrolyte or acid–base balance.

### 3.4 C3 ^−/−^ modulates immune populations and infiltration during IRI

**FIGURE 5:**
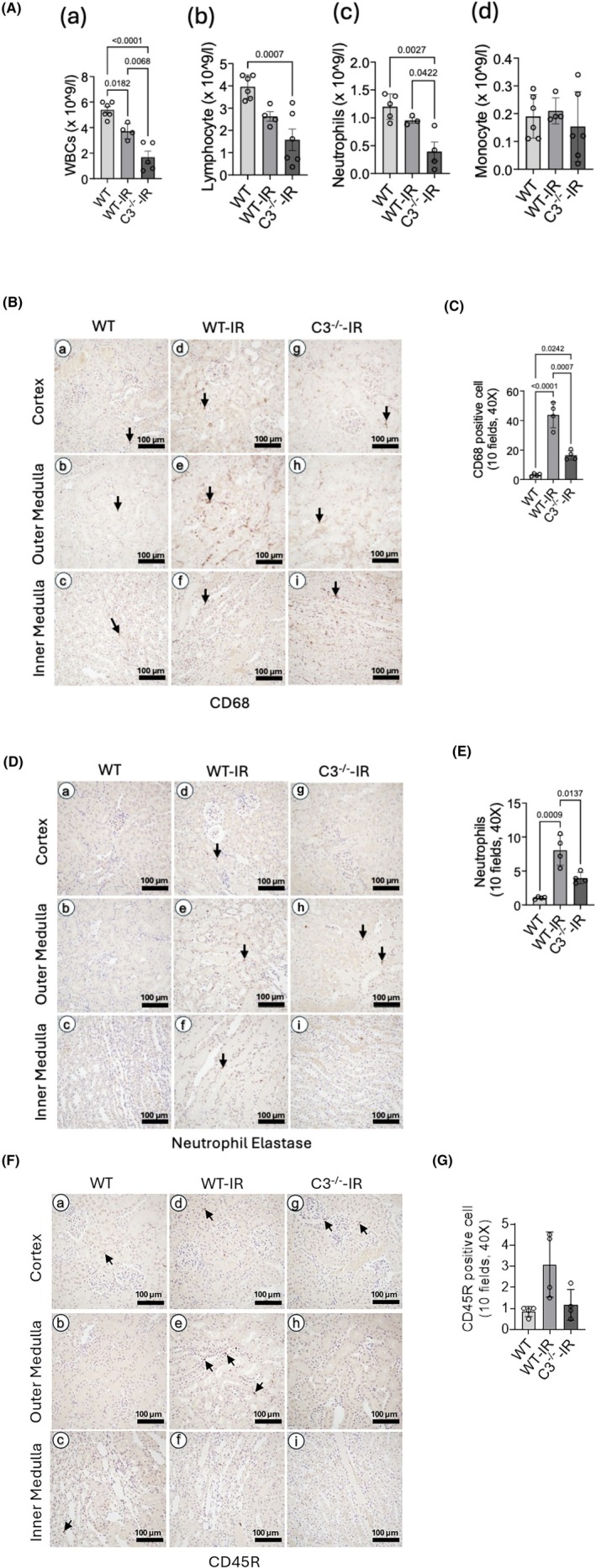
C3 deficiency attenuates systemic leukocyte responses and renal inflammatory cell infiltration following ischemia–reperfusion injury. (A) Quantification of peripheral blood leukocyte populations via a VetScan HM5 analyzer, including (a) total white blood cells (WBCs), (b) lymphocytes, (c) neutrophils, and (d) monocytes. Data are shown as the mean ± SD, *n* = 4–6. (B, D, F) Representative micrographs from formalin‐fixed kidney sections (*n* = 4) stained for (B) CD68+ macrophages, (D) neutrophils, and (F) CD45R+ B cells. Black arrows indicate respective positive cells in kidneys. (C, E, G) Bar graphs showing quantification of respective positive cells per 10 fields (40×) for (C) CD68+ macrophages, (E) neutrophils, and (G) B cells. Data are shown as the mean ± SD, *n* = 4. *p* values for pairwise comparisons are displayed above the corresponding bars.

To examine the impact of C3 deletion on circulating immune cell populations during IRI, blood cell counts were analyzed using a VetScan HM5 analyzer. WT controls displayed robust basal white blood cell (WBC) counts, which were significantly decreased after IRI (Figure 5A a). Notably, C3^−/−^ rats subjected to IRI exhibited an even further decrease in WBC counts, potentially indicating that the absence of C3 disrupts circulating leukocytes (Figure 5A a). A similar pattern was observed for lymphocytes and neutrophils (Figure 5A b,c). In contrast, monocyte counts remained unchanged among all groups (Figure 5A d), suggesting that the effect of C3 deletion during IRI is selective for certain leukocyte populations rather than a global suppression of circulating immune cells.

To assess whether these systemic changes were reflected in immune cell infiltration within the kidney, we performed immunohistochemical staining for CD68 (denoting macrophages), neutrophil elastase (denoting neutrophils), and CD45R (denoting B cells), with respective positive cells indicated using black arrows. WT kidneys displayed basal immune cell infiltration (Figure 5B a–c,D a–c,F a–c) which was exacerbated by IRI, including marked accumulation of CD68^+^ macrophages and neutrophils within the renal cortex and tubulointerstitial regions (Figure 5B d–f,C, D d–f, E). Strikingly, C3^−/−^ rats showed a pronounced reduction in both CD68^+^ macrophages and neutrophil elastase positive cells (Figure 5B g–i,C, D g–i,E) recapitulating prior reports that C3 activation contributes to inflammation post‐IRI (Buelli et al., 2024; Cravedi et al., 2013; Wu et al., 2022). No significant differences were observed in CD45R+ B cell kidney infiltration (Figure 5F,G).

Together, these results demonstrate that C3 contributes to both systemic and local immune responses during IRI. Its absence not only further reduces circulating lymphocytes and neutrophils but also attenuates the infiltration of macrophages and neutrophils into the kidney, likely contributing to the preserved renal morphology and improved functional outcomes observed in C3^−/−^ rats.

### 3.5 Proteomic profiling reveals distinct molecular signatures in C3‐ and C5‐deficient kidneys following IRI

**FIGURE 6:**
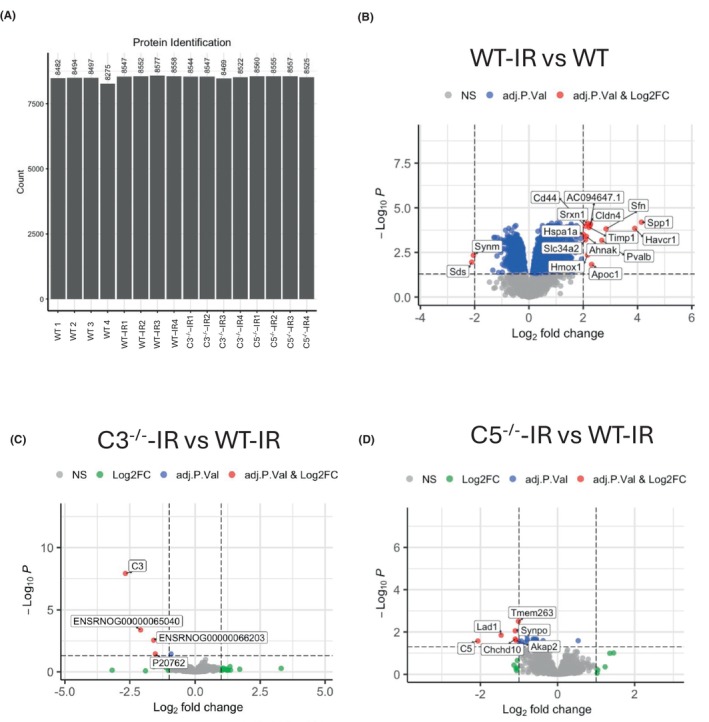
Proteomic profiling reveals C3‐ and C5‐dependent alterations in renal protein expression following ischemia–reperfusion injury. (A) Total number of proteins identified across experimental groups, demonstrating high coverage and reproducibility of nano LC–MS/MS analysis. (B) Volcano plot comparing WT kidneys subjected to ischemia–reperfusion injury (WT‐IR) versus WT controls. Significantly altered proteins are highlighted based on adjusted *p*‐value and log_2_ fold change, including upregulated injury‐associated proteins such as Havcr1 (KIM‐1), Spp1, Sfn, and Hspa1a, and downregulated proteins including Synm and Sds. (C) Volcano plot of C3^−^/^−^‐IR versus WT‐IR kidneys showing selective downregulation of complement C3 and immunoglobulin‐related proteins in the absence of C3. (D) Volcano plot of C5^−^/^−^‐IR versus WT‐IR kidneys illustrating reduced expression of C5 and proteins associated with structural and signaling pathways, including Tmem263, Synpo, Lad1, Chchd10, and Akap2. Dashed lines indicate thresholds for statistical significance and fold change. Data points are color‐coded as non‐significant (NS), significant by adjusted *p*‐value, log_2_ fold change, or both.

Complement activation is a key driver of kidney injury during ischemia–reperfusion (Gorsuch et al., 2012; Mcgraw et al., 2023; McGraw et al., 2025; Zhou et al., 2000); however, the molecular pathways underlying the protective effects of C3 and C5 deficiency remain incompletely understood. To investigate this, we performed nano LC–MS/MS–based proteomic profiling of renal tissue harvested from WT controls, as well as WT, C3^−/−^, and C5^−/−^ rats following IRI. More than 8000 proteins were identified with high reproducibility among biological replicates (Figure 6A). Comparative analysis revealed distinct protein expression patterns in C3‐ and C5‐ deficient kidneys (Figure 6B–D) indicating that targeting the upstream versus downstream complement pathway exerts differential effects on the renal proteome.

Proteomic analysis identified 16 proteins significantly altered from healthy WT controls following IRI (Table 2), including 2 downregulated and 14 upregulated proteins. Among the upregulated proteins, the most prominent were osteopontin (Spp1), hepatitis A virus cellular receptor 1 homolog (Havcr1, also referred to as KIM‐1), and stratifin (Sfn). Most differentially expressed proteins were involved in immune responses or cellular repair mechanisms; for example, Spp1 is a multifunctional protein involved in tissue repair/immune responses which acts as a pro‐inflammatory cytokine (Ding et al., 2024). Havcr1/KIM‐1, as previously discussed, is a kidney injury marker which promotes clearance of dead cells (Han et al., 2002). Similarly, Sfn is a cell cycle checkpoint protein which controls DNA damage responses and maintains epithelial cell structure (Wang et al., 2022). Conversely, synemin (Synm; a cytoskeletal intermediate filament) (UniProt, 2026a) and L‐serine dehydratase/L‐threonine deaminase (Sds) (UniProt, 2026b) were downregulated.

**TABLE 2:** List of the differentially expressed proteins in WT‐IR rats kidney compared to WT healthy controls.

Uniport ID	Gene symbol	Description	Average intensity	Log FC	*p* value	Adjusted *p* value	Trend
G3V9G5	Synm	Synemin	10.3843	−2.047	0.0004	0.0046	↓
P09367	Sds	L‐serine dehydratase/L‐threonine deaminase	10.7399	−2.1091	0.0015	0.0112	↓
P08721	Spp1	Osteopontin	12.8299	4.1451	0	0.0001	↑
O54947	Havcr1	Hepatitis A virus cellular receptor 1 homolog	11.8809	3.9007	0	0.0001	↑
G3V9A3	Sfn	Stratifin	12.3057	2.8438	0	0.0002	↑
Q7TP44	Srxn1	Ab2‐390	11.2627	2.1518	0	0.0001	↑
A0A8I6GHZ0	AC094647.1	NADP‐dependent oxidoreductase domain‐containing protein	12.2812	2.2675	0	0.0001	↑
Q5XIT8	Cldn4	Claudin	12.3906	2.2366	0	0.0001	↑
P26051	Cd44	CD44 antigen	11.8892	2.0761	0	0.0001	↑
P30120	Timp1	Metalloproteinase inhibitor 1	10.6436	2.218	0	0.0001	↑
P0DMW0	Hspa1a	Heat shock 70 kDa protein 1A	14.9187	2.0104	0	0.0003	↑
A0A0G2JUA5	Ahnak	AHNAK nucleoprotein	16.2036	2.1168	0	0.0004	↑
Q9JJ09	Slc34a2	Sodium‐dependent phosphate transport protein 2B	13.1698	2.085	0	0.0006	↑
P02625	Pvalb	Parvalbumin alpha	12.3589	2.683	0	0.0007	↑
P06762	Hmox1	Heme oxygenase 1	11.8913	2.1584	0.0004	0.0045	↑
P19939	Apoc1	Apolipoprotein C‐I	13.9709	2.3082	0.0023	0.015	↑

Four proteins were downregulated following C3^−/−^ compared to the WT group post‐IRI, including the C3 protein itself (Figure 6C). These proteins included Ig‐like domain–containing protein (ENSRNOG00000066203), Ig‐like domain–containing protein (ENSRNOG00000065040), and Ig gamma‐2C chain C region (P20762) as outlined in Table 3. In parallel, comparing C5^−/−^ and WT kidneys after IRI identified 6 differentially expressed proteins, including C5, which were all downregulated (Figure 6D). These proteins included Transmembrane protein 263 (Tmem263), A‐kinase anchor protein 2 (AKAP2), Ladinin‐1‐like (Lad1), Coiled‐coil‐helix‐coiled‐coil‐helix domain containing 10 (Chchd10) and Synaptopodin (Synpo) as detailed in Table 4.

**TABLE 3:** List of the differentially expressed proteins in C3^−/−^‐IR rats kidney compared to WT‐IR.

Uniport ID	Gene symbol	Description	Average intensity	Log FC	*p* value	Adjusted *p* value	Trend
P01026	C3	Complement C3	14.9475	−2.6697	0	0	↓
A0A8I5ZYV4	ENSRNOG00000066203	Ig‐like domain‐containing protein	12.1109	−1.5992	0	0.0027	↓
A0A8I6A7R6	ENSRNOG00000065040	Ig‐like domain‐containing protein	11.908	−2.1035	0	0.0004	↓
P20762	P20762	Ig gamma‐2C chain C region	14.0963	−1.537	0	0.0357	↓

**TABLE 4:** List of the differentially expressed proteins in C5^−/−^‐IR rats kidney compared to WT‐IR.

Uniport ID	Gene symbol	Description	Average intensity	Log FC	*p* value	Adjusted *p* value	Trend
A0A8I6AQR3	Tmem263	Transmembrane protein 263	10.0994	−1.0146	0	0.0031	↓
Q9Z327	Synpo	Synaptopodin	11.9014	−1.0973	0	0.0088	↓
A0A8I6GI64	Lad1	Ladinin‐1‐like	12.885	−1.4629	0	0.0141	↓
P08650	C5	Complement C5	10.426	−2.0652	0.0001	0.0266	↓
Q63ZY8	Chchd10	Coiled‐coil‐helix‐coiled‐coil‐helix domain containing 10	10.6398	−1.0966	0	0.0215	↓
Q5U301	Akap2	A‐kinase anchor protein 2	13.0112	−1.0756	0.0001	0.0248	↓

Notably, comparative analysis revealed an inverse expression pattern for a subset of proteins, with proteins reduced in C3^−/−^‐IR showing increased abundance in C5^−/−^‐IR (Table S1).

Overall, proteins that were differentially expressed following complement deficiency participated in immune regulation, cellular signaling, and cytoskeletal organization. These findings suggest that complement activation at different cascade levels exerts differential control over downstream IRI‐associated molecular pathways. Together, these complement‐dependent proteomic alterations may contribute to observed differences in renal injury severity following ischemia–reperfusion.

### 3.6 Loss of C3/C5 during IRI differentially regulates metabolic, immune, and extracellular matrix programs

To understand the pathways associated with identified differentially expressed genes, we performed Gene Ontology (GO) and GSEA pathway enrichment analysis. Due to the limited number of proteins passing multiple‐testing correction, pathway analysis was performed using proteins selected based on unadjusted *p* values and an absolute fold change threshold. False discovery rate (FDR)‐adjusted *p*‐values were calculated but not used as a strict cutoff for enrichment analysis. Accordingly, pathway enrichment analyses are considered exploratory.

**FIGURE 7:**
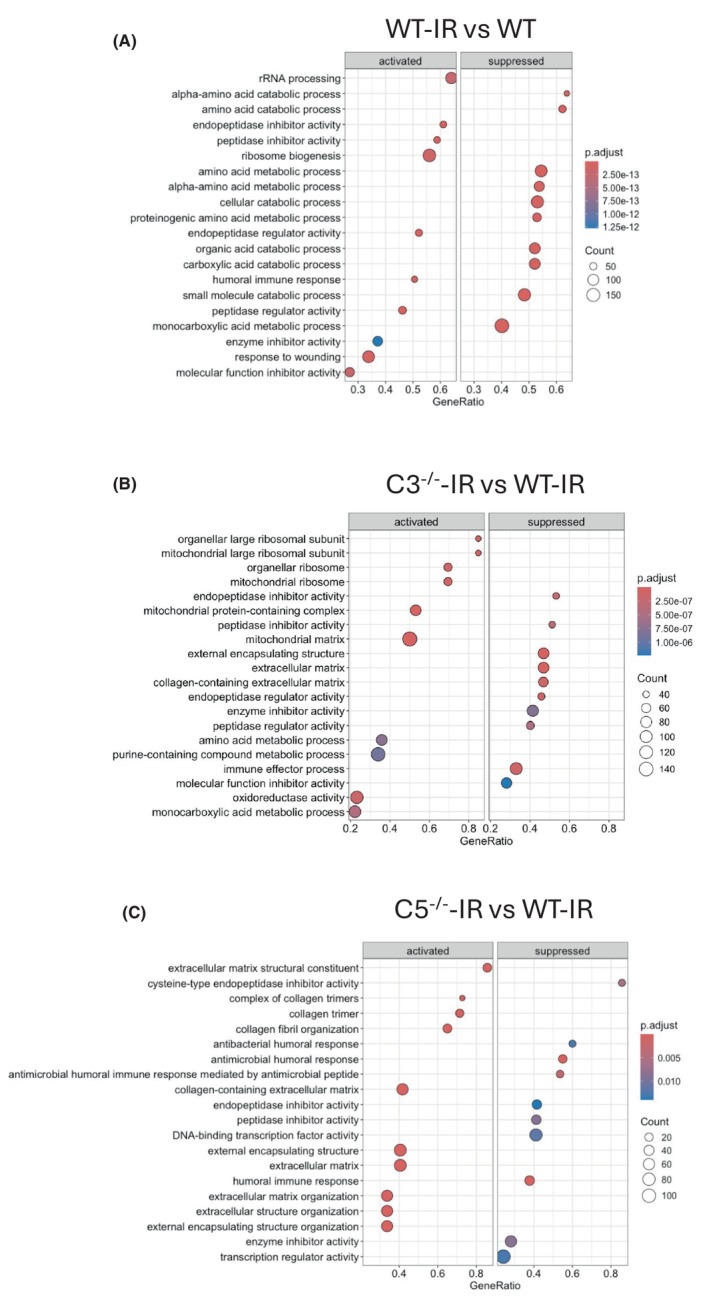
Gene Ontology (GO) enrichment analysis reveals complement‐dependent pathway remodeling following renal ischemia–reperfusion injury. (A) GO enrichment analysis comparing WT kidneys subjected to ischemia–reperfusion injury (WT‐IR) versus WT controls. IRI induced activation of protease regulatory pathways, including endopeptidase inhibitor and regulator activity, alongside humoral immune responses. In contrast, metabolic processes, particularly amino acid and small‐molecule catabolism, were suppressed, indicating early metabolic disruption following injury. (B) GO enrichment analysis of C3^−^/^−^‐IR versus WT‐IR kidneys demonstrates a shift toward mitochondrial and metabolic programs, including activation of mitochondrial ribosomal components, oxidative metabolism, and small‐molecule metabolic processes. Concurrently, pathways related to immune effector function, extracellular matrix organization, and endopeptidase regulation were suppressed, consistent with attenuation of upstream complement‐driven inflammatory responses. (C) GO enrichment analysis of C5^−^/^−^‐IR versus WT‐IR kidneys reveals enrichment of extracellular matrix (ECM)‐associated pathways, including collagen trimer formation, extracellular structure organization, and collagen fibril assembly. At the same time, immune‐related pathways, including humoral and antimicrobial responses, were suppressed, suggesting that downstream complement inhibition favors structural remodeling while dampening inflammation. Dot size represents the number of proteins contributing to each term, and color indicates adjusted *p*‐value significance. Gene ratio reflects the proportion of proteins associated with each GO term. Activated and suppressed pathways are displayed separately.

Compared to WT controls, renal IRI triggered protease regulatory programs, including activation of endopeptidase inhibitor and regulator pathways (Figure 7A). At the same time, amino acid and small‐molecule catabolic processes were suppressed (Figure 7A). This suggests that, early after injury, protective protease regulation is increased but certain metabolic processes are disrupted.

**FIGURE 8:**
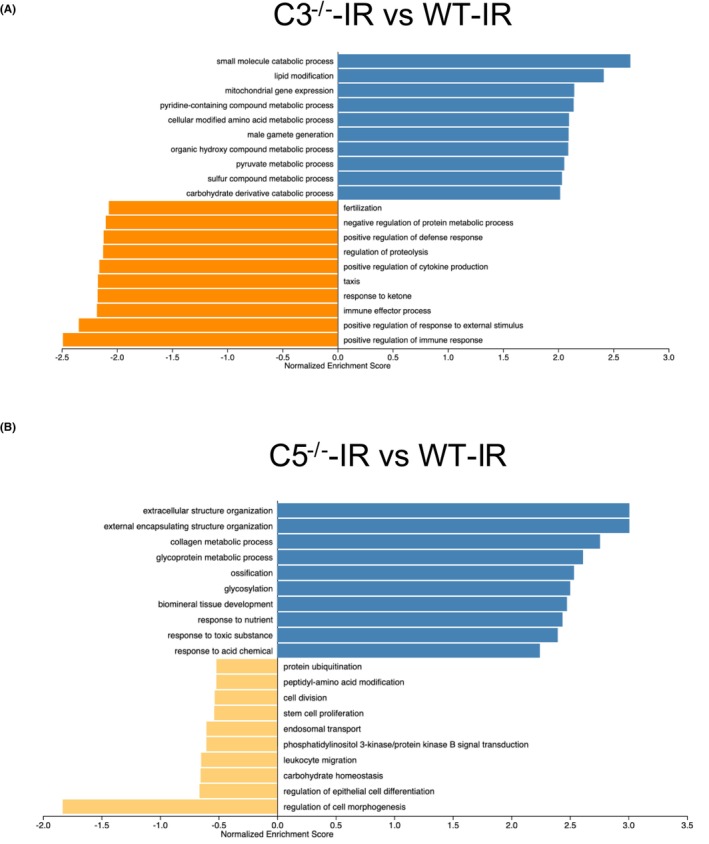
Gene set enrichment analysis (GSEA) reveals distinct biological programs regulated by complement C3 and C5 deficiency following renal ischemia–reperfusion injury. (A) GSEA comparing C3^−^/^−^‐IR versus WT‐IR kidneys demonstrates significant enrichment of metabolic and mitochondrial pathways, including small‐molecule catabolism, lipid modification, and mitochondrial gene expression. In contrast, immune and inflammatory pathways—such as cytokine production, immune effector processes, and response to external stimuli—are negatively enriched, indicating suppression of upstream complement‐driven immune activation. (B) GSEA comparing C5^−^/^−^‐IR versus WT‐IR kidneys reveals strong enrichment of extracellular matrix (ECM) and structural remodeling pathways, including extracellular structure organization, collagen metabolism, and glycoprotein processing. Conversely, pathways related to cell proliferation, intracellular signaling, and leukocyte migration are negatively enriched, suggesting reduced immune activation and altered cellular dynamics following downstream complement inhibition. Normalized enrichment score (NES) reflects the degree of pathway enrichment, with positive values indicating activation and negative values indicating suppression.

Notably, C3^−/−^ markedly shifted activated pathways toward metabolic and mitochondrial activity compared to WT post‐IRI. This included organellular/mitochondrial ribosome machinery and metabolic processes (Figure 7B). At the same time, pathways related to immune effector function, cytokine production, endopeptidase inhibition, and extracellular matrix regulation were suppressed, indicating a dampened immune and extracellular response in the absence of C3 (Figure 7B). GO analysis of the C3^−/−^ IRI group identified small molecule catabolism, lipid modification, mitochondrial gene expression, and purine metabolism as enriched biological processes compared to WT (Figure 8A).

By contrast, comparison of C5^−/−^ to WT after IRI produced identified activation of pathways involved in extracellular matrix structure and collagen trimer/fibril organization (Figure 7C), corroborated by GO enrichment of collagen metabolism and extracellular/external encapsulating structure organization (Figure 8B). At the same time, immune‐related processes‐including humoral responses, leukocyte migration, and epithelial differentiation were suppressed, suggesting that loss of C5 favors structural remodeling and reduces immune activation post‐IRI (Figures 7C and 8B).

Together, these analyses reveal that C3 and C5 shape the kidney's response to injury. Upstream C3 deficiency enhances mitochondrial and metabolic pathways while suppressing immune and extracellular signals. Downstream C5 deficiency also suppresses immune responses but promotes extracellular structure remodeling which may be crucial to wound healing. These findings highlight how different complement components orchestrate metabolic, immune, and structural adaptations during IRI, providing a nuanced view of their roles in tissue repair and injury responses.

### 3.7 Validation of proteomic findings by immunohistochemistry and Western blot

**FIGURE 9:**
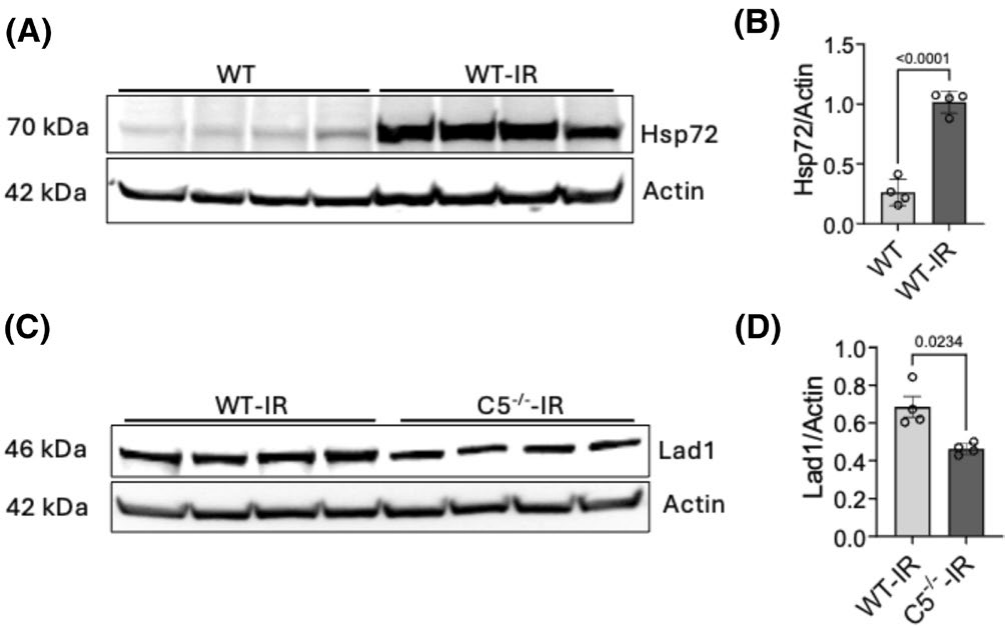
Western blot validation of proteomic findings following renal ischemia–reperfusion injury. (A) Representative immunoblots and (B) densitometric quantification of Hsp72 (Hspa1a) expression in kidneys. (C) Representative immunoblots and (D) densitometric analysis of Lad1 expression in kidneys. Actin was used as a loading control for normalization. Data are presented as mean ± SD (*n* = 4 per group). *p* values for pairwise comparisons are displayed above the corresponding bars. Molecular weight (kDa) positions are indicated on the immunoblots. Representative cropped blot images are shown for clarity; full, uncropped blots with molecular weight markers are provided in Figures S5–S8.

Mass spectrometry findings were validated using complementary experimental approaches. Hspa1a (Hsp72), which was significantly increased in WT‐IR compared with WT controls in the proteomic dataset (Table 2), was confirmed by Western blot analysis to be similarly increased (Figure 9A,B).

In the C3^−/−^ model, C3 was selected for validation due to the availability of a specific antibody among the identified proteins. Its downregulation in C3^−/−^ kidneys following IRI was confirmed by both Western blot and immunohistochemistry (Figure 1D–G), validating the proteomic findings (Table 3). Similarly, in the C5^−/−^ model, Western blot analysis further supported the proteomic data by demonstrating significantly reduced expression of Lad1 in C5^−^/^−^ rats compared with WT following IRI (Figure 9C,D). This was consistent with mass spectrometry results (Table 4).

Collectively, these validation experiments reinforce the proteomic findings and confirm differential expression of injury‐associated proteins across WT, C3^−/−^, and C5^−/−^ models following IRI.

## 4 DISCUSSION

In this study, we utilized C3^−/−^ and C5^−/−^ transgenic rodent models to delineate the relative contributions of upstream and downstream complement components in renal IRI. Similar to our group's previous study in C5^−/−^ rodents post‐IRI (McGraw et al., 2025), C3 deficiency conferred robust protection against renal dysfunction, tubular necrosis, and immune cell infiltration. Together, these data demonstrate that inhibition of either upstream or downstream complement components represents a viable strategy to mitigate renal injury. By integrating functional assessments with quantitative proteomics, we identified distinct molecular programs driven by deletion of either C3 or C5 during IRI, providing mechanistic insights into how complement orchestrates renal injury and repair.

C3 occupies a central position in the complement cascade, integrating signals from the classical, lectin, and alternative pathways and generating effector molecules including C3a (an “anaphylatoxin”) and C3b, an essential component of the C5 convertase enzyme (Cravedi & Heeger, 2014; Howard et al., 2021; Wei et al., 2025). Activation of the C5 convertase initiates the terminal complement pathway through cleavage of C5 (Azoulay et al., 2024). Because C3 deficiency limits downstream C5 activation, C3^−/−^ rats displayed protection from IRI‐induced renal dysfunction and tubular injury/necrosis comparable to that observed in C5^−/−^ rats in our prior work (McGraw et al., 2025). These findings align with previous studies reporting reduced functional injury following pharmacological or genetic inhibition of C3 (Wu et al., 2022; Zheng et al., 2006). The observation that C3^−/−^ and C5^−/−^ exhibit similar degrees of protection suggests inhibition of upstream complement activation may not be required to achieve the desired therapeutic benefit in acute renal injury.

The activation of C3/C5 produces anaphylatoxins C3a and C5a, which act as ligands for immune cell receptors and drive cellular activation, differentiation, and chemotaxis (Buelli et al., 2024; Laumonnier et al., 2020). Both anaphylatoxins are central mediators of inflammation in renal IRI and contribute to acute damage that can progress to fibrosis (Arumugam et al., 2003; Peng et al., 2012; Wu et al., 2023). C5a is at least 100‐fold more potent than C3a as a chemotactic factor (Chenoweth, 2026), consistent with our previous observation that C5^−^/^−^ rats exhibit markedly reduced renal infiltration of macrophages, neutrophils, and B cells without changes in circulating leukocyte counts (McGraw et al., 2025). Although C3^−/−^ similarly reduced immune cell infiltration (likely due to disrupted C5a production), we additionally observed reduced circulating WBCs in C3^−/−^ animals, suggesting a role for C3 in leukocyte survival or maintenance. This is supported by prior studies demonstrating that C3 deficiency impairs cell survival pathways, including metabolic reprogramming (Arbore et al., 2017; Elvington et al., 2016; Ratajczak et al., 2025; Sahu, 2023), highlighting noncanonical roles for complement proteins beyond classical immune functions.

To further elucidate molecular pathways altered by complement deficiency, we compared renal proteomes of WT, C3^−/−^, and C5^−/−^ rats post‐IRI. Although IRI itself induced upregulation of 14 proteins associated with tissue repair/immune responses relative to healthy WT controls, complement‐dependent proteomic changes were more selective. C3 deficiency resulted in downregulation of four proteins, including those containing Ig‐like domains (Barclay, 1999), all of which are integral to immune function and consistent with the broad immunomodulatory effects of upstream complement inhibition. In contrast, C5 deficiency selectively downregulated proteins associated with cytoskeletal organization and scaffolding (AKAP2, Lad1, Synpo) (Kremerskothen et al., 2005; Maric et al., 2021; Wang et al., 2025), mitochondrial cristae formation (Chchd10) (Cell Death & Disease, 2026), and growth hormone signaling (Tmem263) (Sarver et al., 2024). Although the membrane attack complex (MAC) is known to interact with cytoskeletal elements during IRI (Cybulsky et al., 2004), broader roles for C5 in regulating cellular architecture and growth remain unexplored. These findings warrant further investigation into how upstream versus downstream complement signaling intersects with fundamental cellular processes, such as metabolism and cytoskeletal dynamics.

Despite the modest number of differentially expressed proteins, exploratory pathway enrichment analyses revealed coherent functional patterns consistent with the observed physiological and histological outcomes. C3^−/−^ kidneys exhibited enrichment of pathways involved in mitochondrial gene expression, lipid modification, and small‐molecule catabolism, alongside suppression of immune effector pathways. In contrast, C5^−/−^ kidneys showed upregulation of extracellular matrix organization and structural remodeling pathways, while immune‐related pathways were similarly downregulated. These signatures highlight potential nonredundant roles for C3 and C5 in shaping renal responses to ischemic injury. Our findings are consistent with prior renal IRI proteomic studies identifying dysregulation of energy metabolism, oxidative stress, and cytoskeletal integrity (Chen et al., 2023; Huang et al., 2018; Li et al., 2024; Luo et al., 2026). However, it is important to note that with a small number of significantly altered proteins, the pathway analysis conducted within this article is exploratory and requires additional validation in future studies.

Collectively, these results provide a nuanced framework for complement‐targeted therapies in AKI and other complement‐mediated diseases. Broad inhibition of complement activation may inadvertently impair beneficial immune or metabolic adaptations, whereas selective targeting of downstream components such as C5 may offer therapeutic efficacy while preserving protective pathways.

### 4.1 Limitations of the studies

Several limitations should be considered when interpreting these findings. First, our proteomic analyses assessed steady‐state protein abundance and did not directly evaluate protein activity or function; future studies incorporating targeted functional assays are needed to validate pathway activation identified in our exploratory analysis. In addition, proteomic profiling was limited to early post‐IRI time points, and the long‐term consequences of C3 or C5 deficiency on renal repair, fibrosis, and chronic disease progression remain unclear. This is particularly relevant given the observed potential effects on cellular metabolism, cytoskeletal organization, and extracellular matrix regulation—processes integral to wound healing.

Another important limitation is the use of constitutive transgenic rodent models, rather than pharmacological inhibition of C3/C5. While genetic deletion ensures complete and sustained loss of C3 or C5, which is often difficult to achieve pharmacologically (Nishimura et al., 2014), it also introduces the possibility that lifelong complement deficiency results in adaptive cellular changes not representative of acute therapeutic inhibition. Furthermore, these potential adaptive changes may have broader effects on the immune response post‐IRI, influencing immune cell trafficking to the kidney. For this reason, we have included analysis of immune cells not only in the circulation but also within renal tissue post‐IRI. Interpretation is further complicated by the fact that C3 deletion inherently suppresses downstream C5 activation. Future work should aim to disentangle C3‐specific effects from C5‐mediated processes, including analysis of intracellular versus circulating complement activity.

### 4.2 Future directions

Our study provided a systems‐level insight into protein networks regulated by upstream (C3) or downstream (C5) complement components during IRI. These findings have translational relevance, as multiple C3‐ and C5‐targeted therapies (Garg & Frishman, 2023; Kuehn, 2025; Legendre et al., 2013; Novartis, 2026; PCORI, 2026; Stern & Connell, 2019) are currently FDA‐approved for clinical use. Future studies should focus on defining the mechanisms by which C3 and C5 regulate cellular metabolism, cytoskeletal integrity, and extracellular matrix dynamics, as well as delineating the role of intracellular complement activation and downstream effector molecules such as anaphylatoxins.

## 5 CONCLUSIONS

In summary, blockade of distinct nodes within the complement cascade elicits divergent molecular and cellular responses during renal IRI. C3 deficiency preferentially enhances mitochondrial and metabolic pathways while suppressing immune signaling, whereas C5 deficiency promotes extracellular matrix remodeling and attenuates humoral immune responses. These findings advance our mechanistic understanding of complement biology in the kidney and suggest that selective modulation of C3 or C5 may enable precision therapeutic strategies for mitigating ischemic renal injury.

## AUTHOR CONTRIBUTIONS

**Dinesh Bhattarai:** Data curation; formal analysis; investigation; methodology; validation; visualization. **Amod Sharma:** Data curation; formal analysis; investigation; methodology; validation; visualization. **Madison McGraw:** Investigation; methodology. **Se‐Ran Jun:** Data curation; formal analysis; validation. **Neelam Joshi:** Methodology; visualization. **Neriman Gokden:** Formal analysis; methodology; validation; visualization. **Samuel Mackintosh:** Data curation; methodology. **Nirmala Parajuli:** Conceptualization; data curation; formal analysis; funding acquisition; investigation; project administration; resources; supervision; validation.

## FUNDING INFORMATION

This research was funded by the National Institute of Health, NIKKD R01 DK123264 (Parajuli), and the APC was funded by Department of Surgery, Division of Transplant Surgery, (Bhattarai), University of Utah School of Medicine, Salt Lake City, Utah.

## CONFLICT OF INTEREST STATEMENT

The authors declare no conflicts of interest.

## ETHICS STATEMENT

The animal care protocol and procedures in the study were approved by the University of Arkansas for Medical Sciences Institute Institutional Animal Care and Use Committee (Protocol code number: 4099; approved on 11 May 2021) and followed the guidelines of the National Institute of Health Guide for the Care and Use of Laboratory Animals.

## Supporting information


Data S1: Western blot images.

**Table S1:** List of the differentially expressed proteins in C5^−/−^‐IR rats kidney compared to C3^−/−^‐IR.

## Data Availability

All data associated with this study are present in the paper. MS data have been deposited in PRIDE and ProteomeXchange (http://www.ebi.ac.uk/pride). PRIDE is a part of ProteomeXchange as shown on the website (https://www.proteomexchange.org/) The identifier (project accession) PXD077461 can be used to access data via ProteomeXchange.
