# Supplementary material for: Effect of complement 3/5 knockout on renal proteomics landscape after ischemia and reperfusion injury in rats
Source: Physiol Rep. 2026 Jul 20;14(14):e71017. doi: 10.14814/phy2.71017 (PMC13385224; doi:10.14814/phy2.71017)
Supplement: Supplementary file 1 — Data S1: Western blot images. [file PHY2-14-e71017-s001.pptx]

## Slide 1
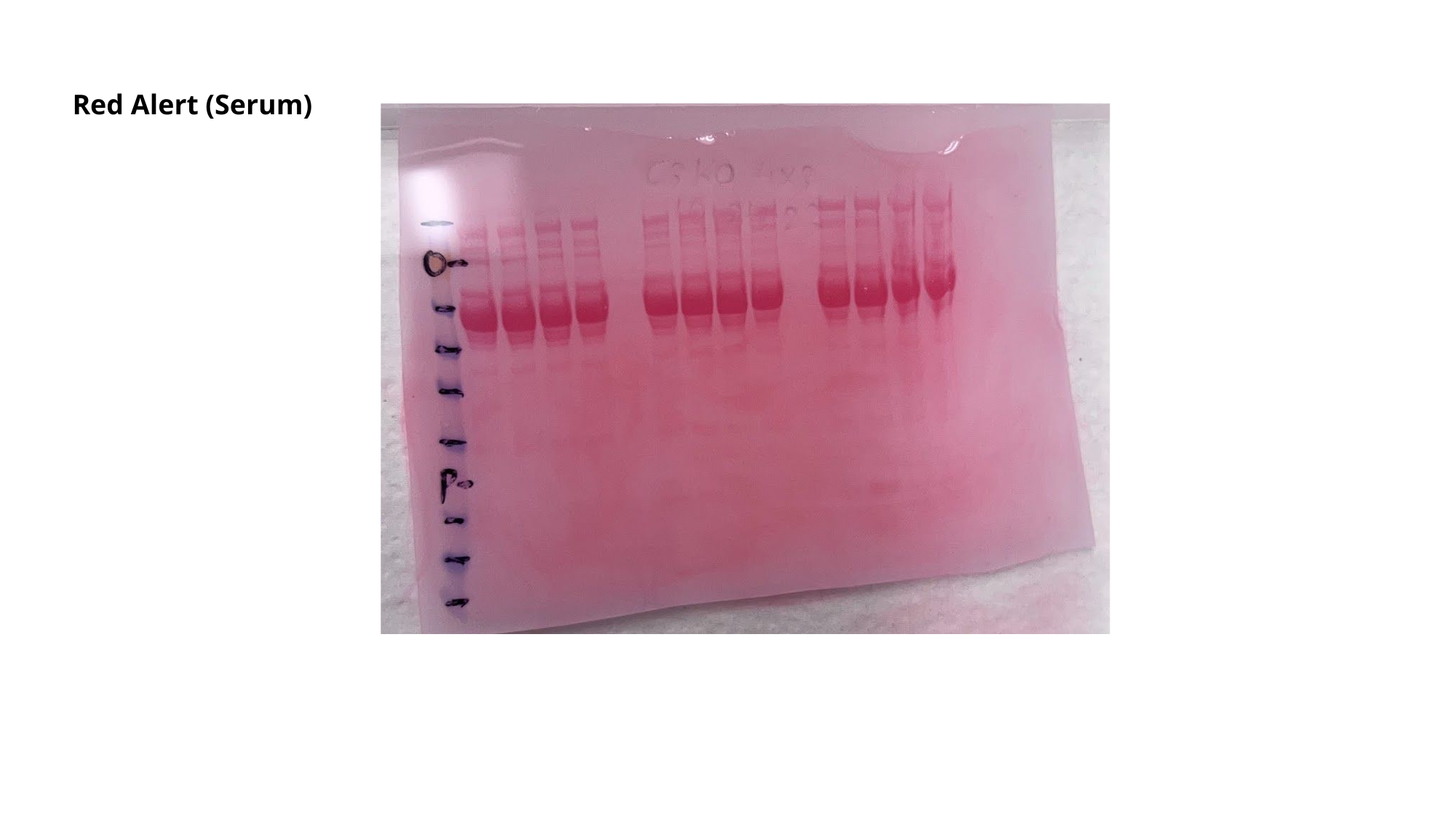

Red Alert (Serum)

## Slide 2
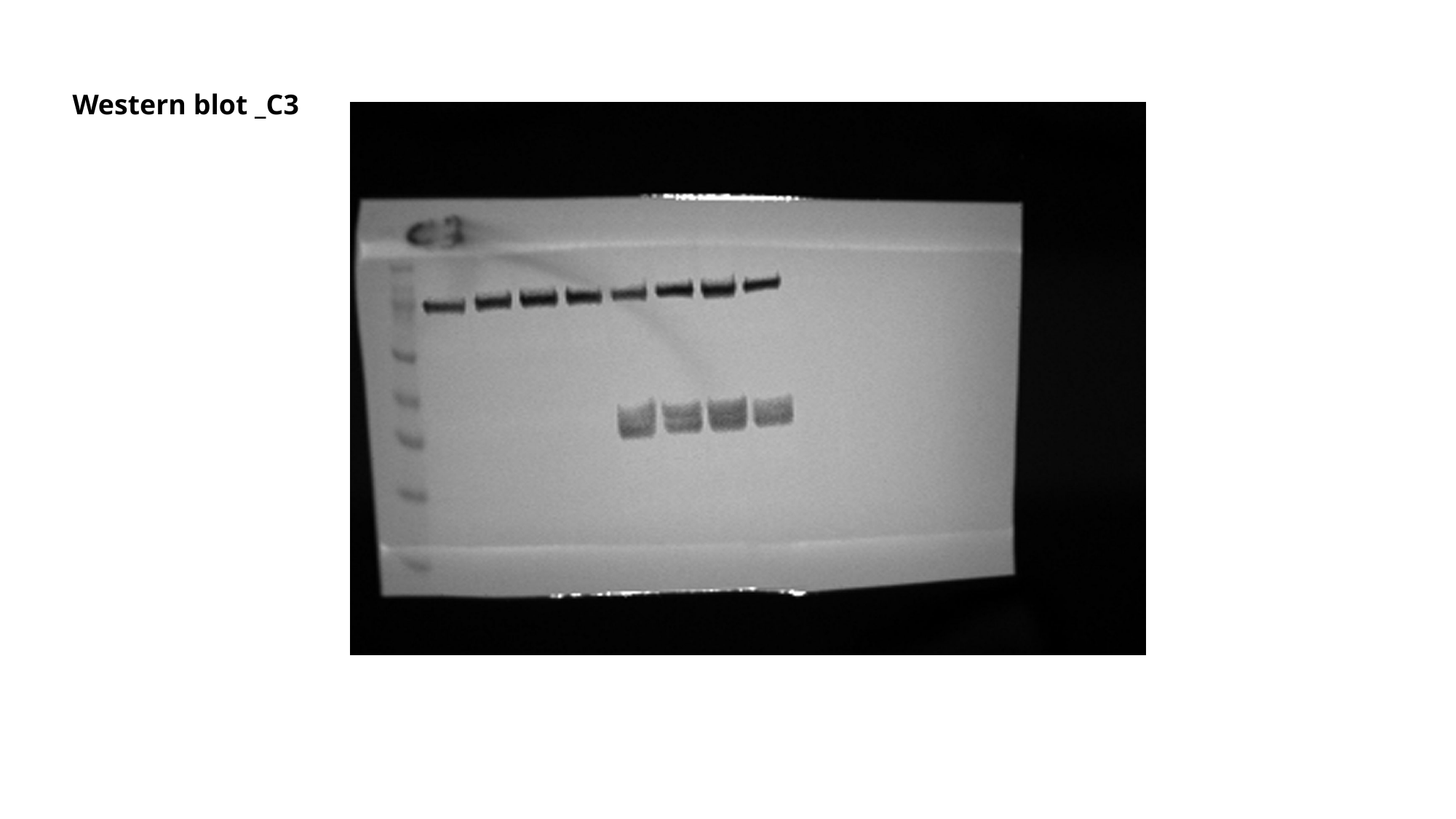

Western blot _C3

## Slide 3
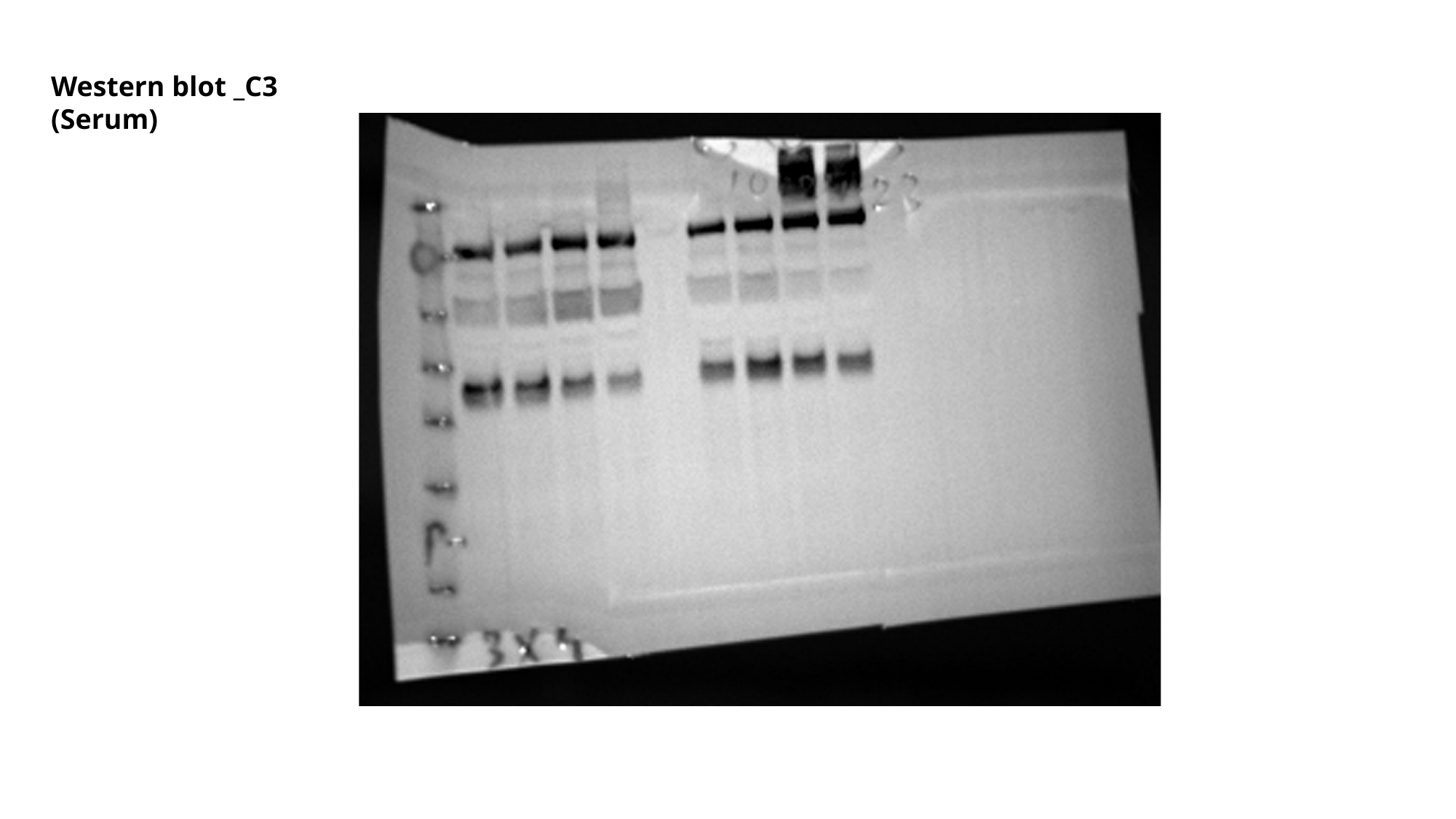

Western blot _C3 (Serum)

## Slide 4
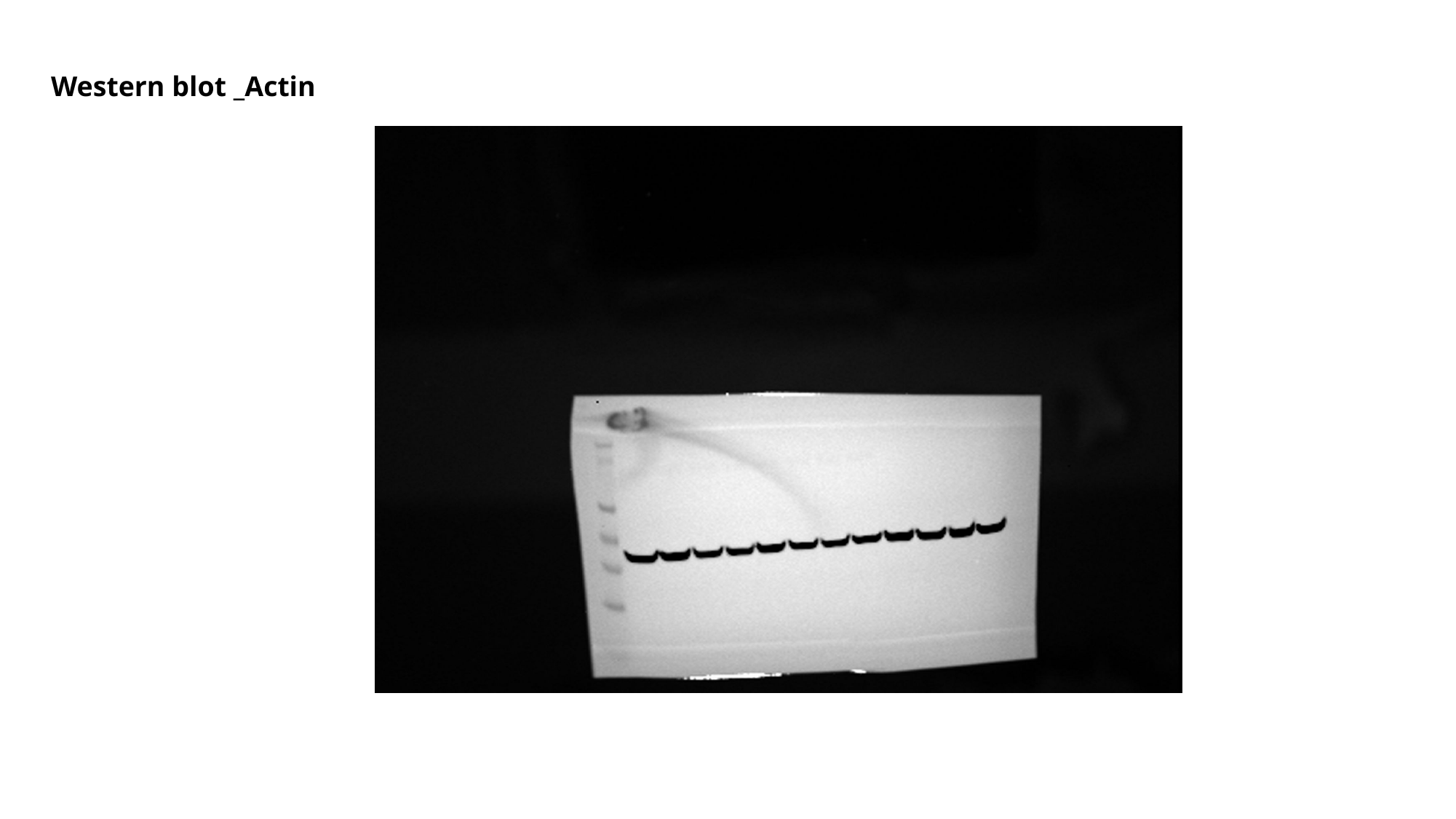

Western blot _Actin

## Slide 5
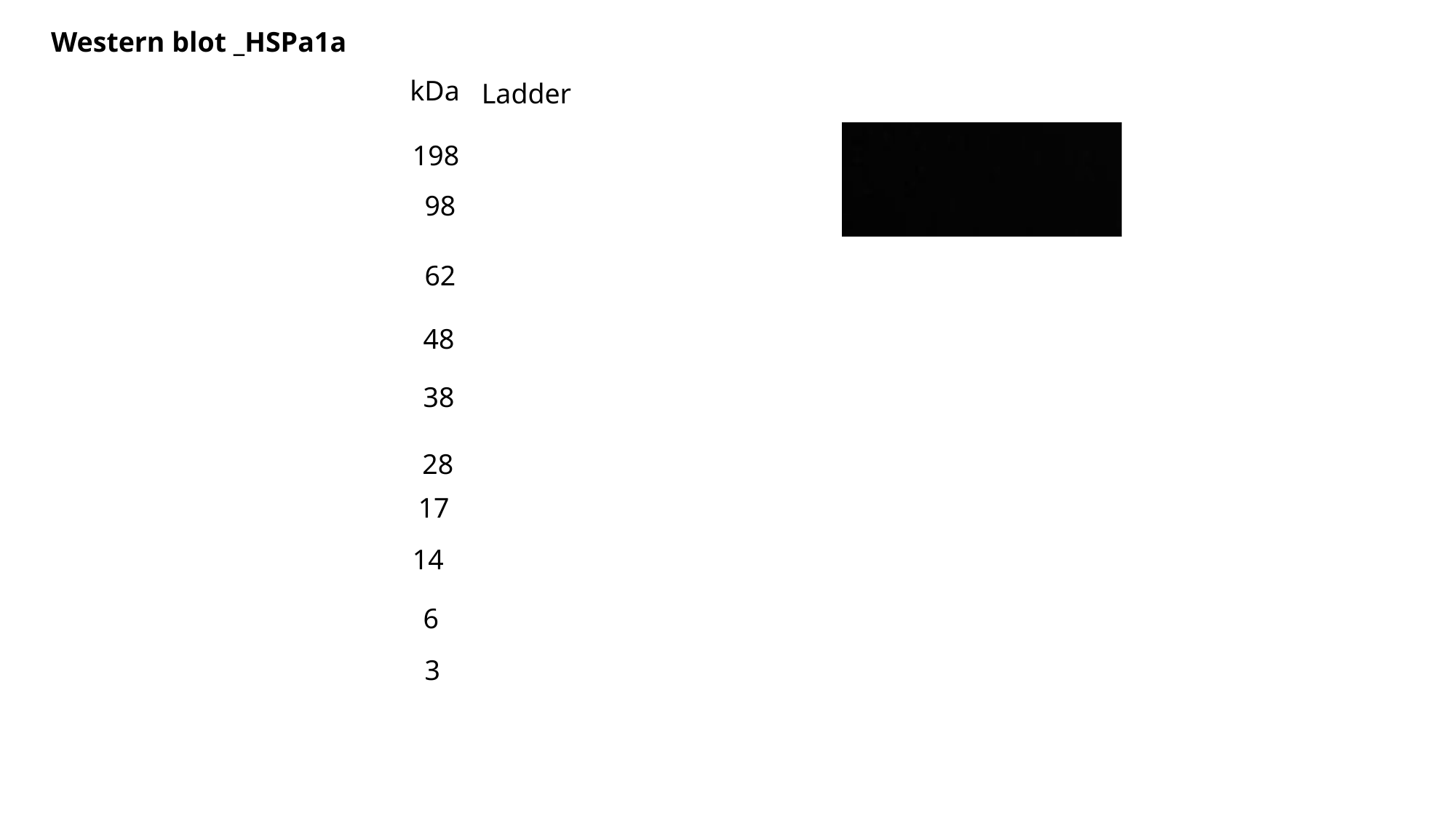

Western blot _HSPa1a
kDa
Ladder
198
98
62
48
38
28
17
14
6
3

## Slide 6
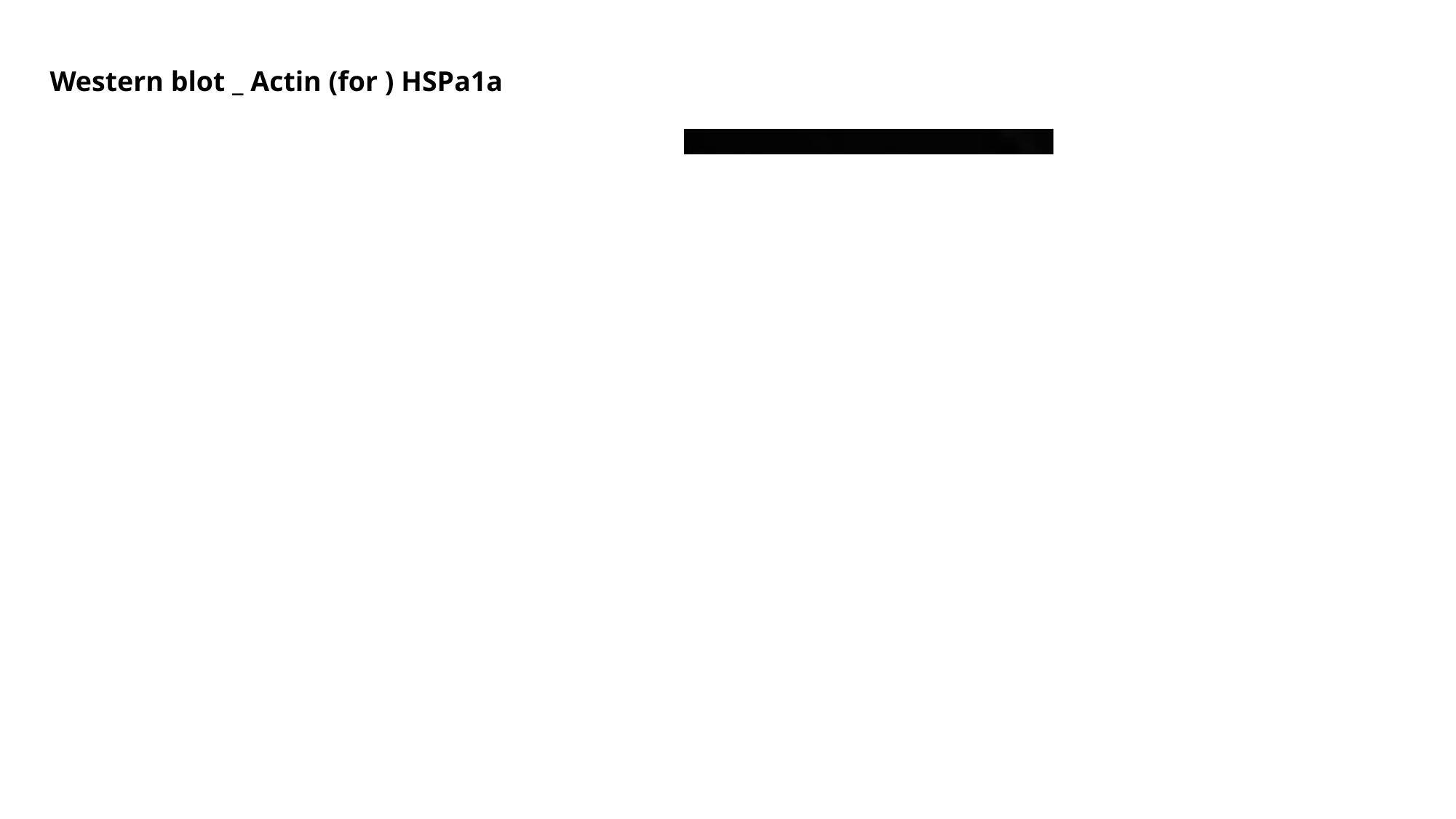

Western blot _ Actin (for ) HSPa1a

## Slide 7
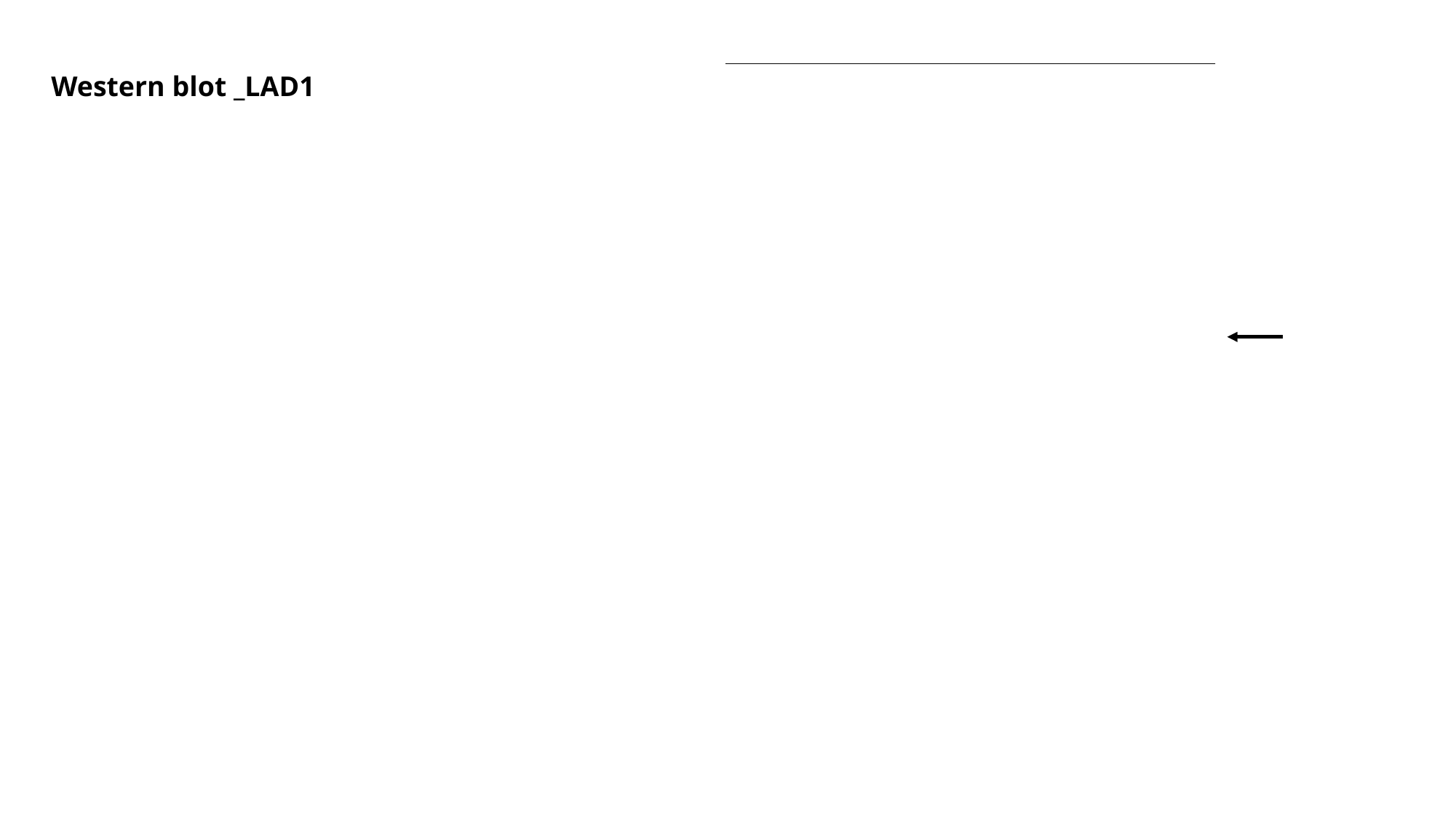

Western blot _LAD1

## Slide 8
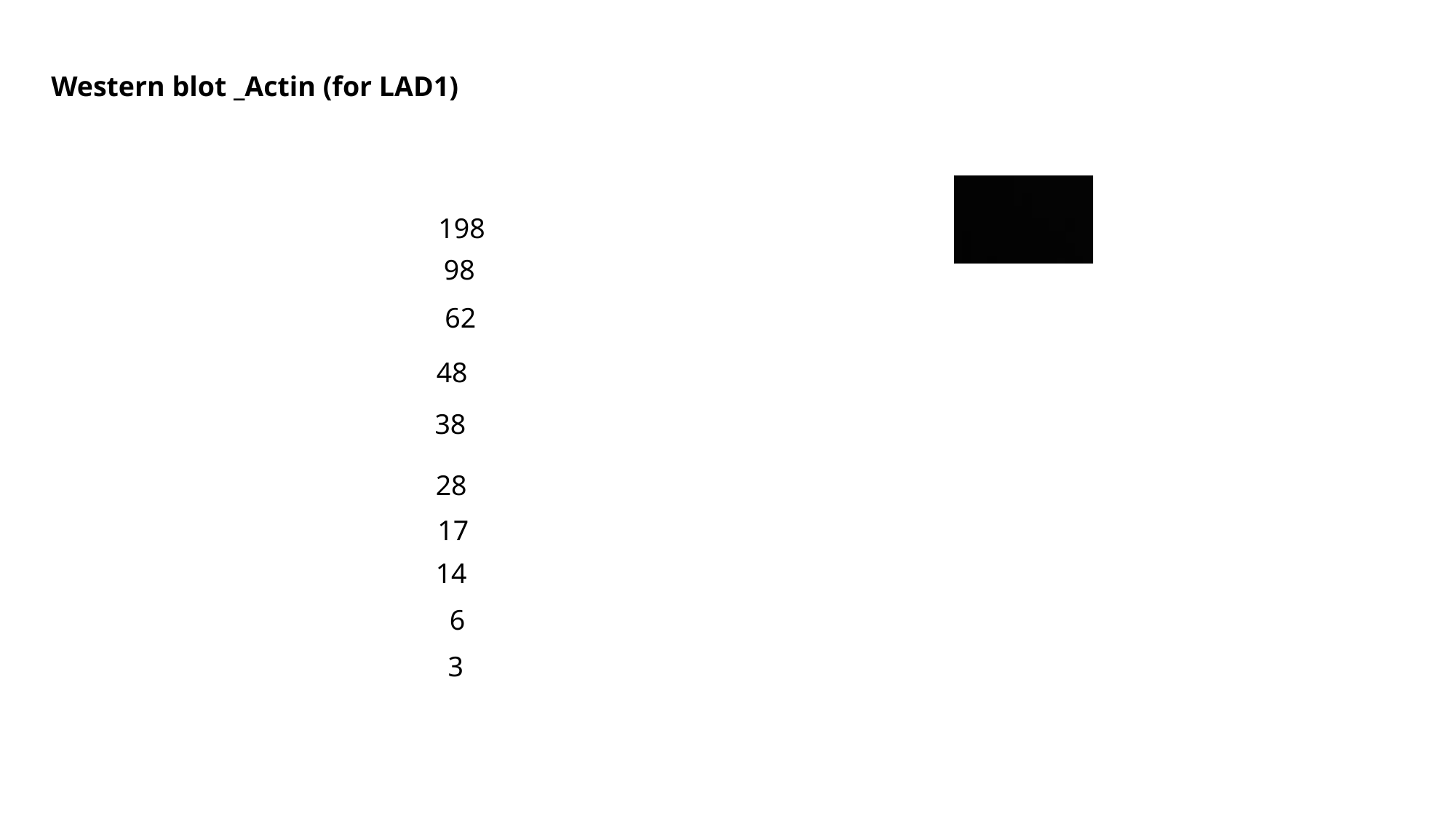

Western blot _Actin (for LAD1)
198
98
62
48
38
28
17
14
6
3

## Slide 9
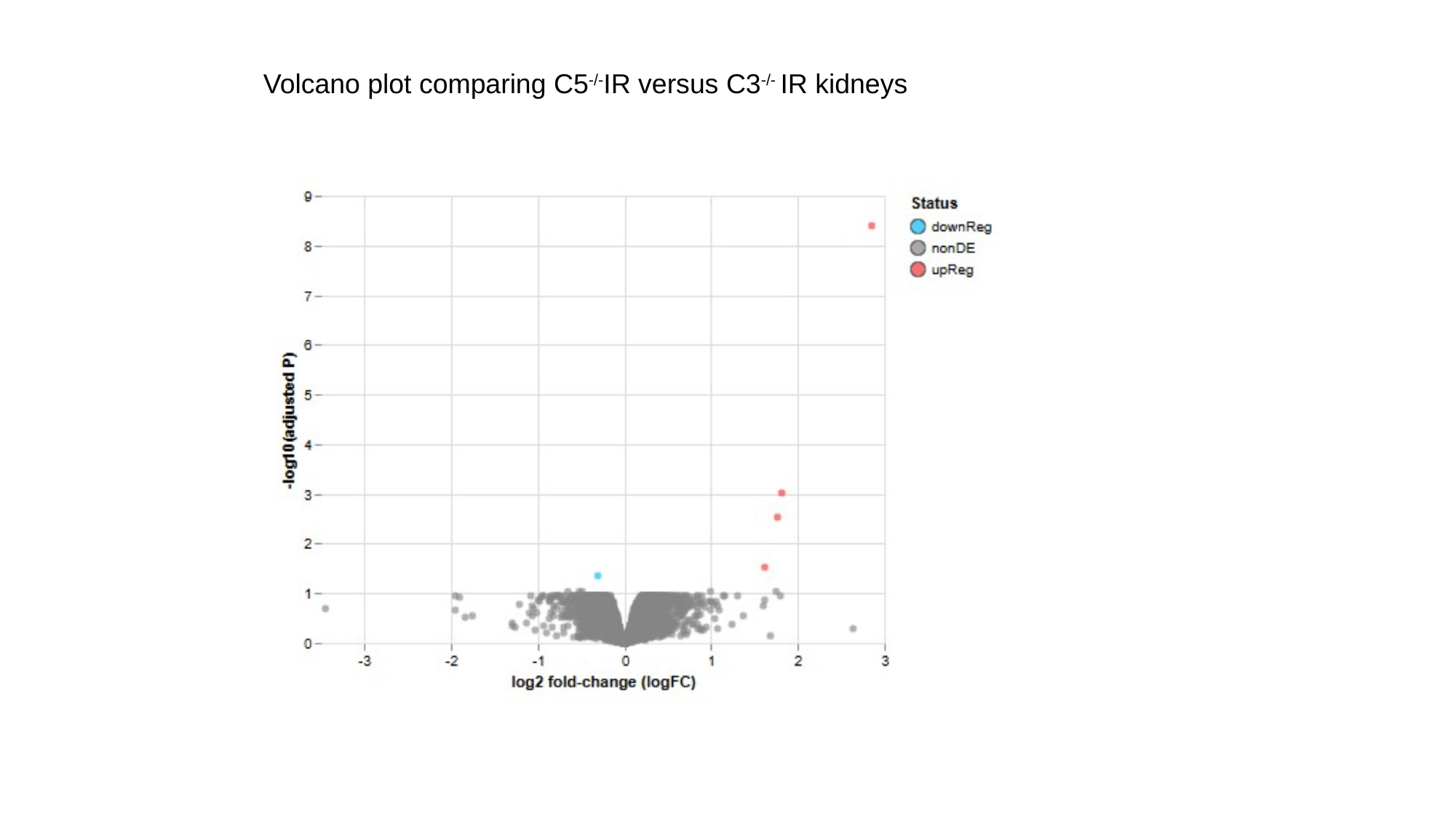

Volcano plot comparing C5-/-IR versus C3-/- IR kidneys
