# Supplementary material for: Effect of complement 3/5 knockout on renal proteomics landscape after ischemia and reperfusion injury in rats
Source: Physiol Rep. 2026 Jul 20;14(14):e71017. doi: 10.14814/phy2.71017 (PMC13385224; doi:10.14814/phy2.71017)
Supplement: Supplementary file 2 — Table S1: List of the differentially expressed proteins in C5−/−‐IR rats kidney compared to C3−/−‐IR. [file PHY2-14-e71017-s002.docx]

**Table SI 1:** List of the differentially expressed proteins in C5^-/-^-IR rats kidney compared to C3^-/-^-IR

| **Uniport ID** | **Gene Symbol** | **Description** | **Average Intensity** | **Log FC** | **P value** | **Adjusted p value** | **Trend** |
| --- | --- | --- | --- | --- | --- | --- | --- |
| P01026 | C3 | Complement C3 | 14.9475 | 2.8566 | 0 | 0 |  |
| A0A8I5ZYV4 | ENSRNOG00000066203 | Ig-like domain-containing protein | 12.1109 | 1.8177 | 0 | 0.0009 |  |
| A0A8I6A7R6 | ENSRNOG00000065040 | Ig-like domain-containing protein | 11.908 | 1.7708 | 0 | 0.003 |  |
| P20762 | P20762 | Ig gamma-2C chain C region | 14.0963 | 1.612 | 0 | 0.0298 |  |
| Q9WTPO | Epb41l1 | Band 4.1-like protein 1 | 12.9517 | -0.3082 | 0 | 0.044 |  |
